# Measurements of jet multiplicity and jet transverse momentum in multijet events in proton–proton collisions at ${\sqrt{s}=13\, \text {TeV}}$

**DOI:** 10.1140/epjc/s10052-023-11753-y

**Published:** 2023-08-22

**Authors:** A. Tumasyan, W. Adam, J. W. Andrejkovic, T. Bergauer, S. Chatterjee, K. Damanakis, M. Dragicevic, A. Escalante Del Valle, R. Frühwirth, M. Jeitler, N. Krammer, L. Lechner, D. Liko, I. Mikulec, P. Paulitsch, F. M. Pitters, J. Schieck, R. Schöfbeck, D. Schwarz, S. Templ, W. Waltenberger, C. -E. Wulz, M. R. Darwish, E. A. De Wolf, T. Janssen, T. Kello, A. Lelek, H. Rejeb Sfar, P. Van Mechelen, S. Van Putte, N. Van Remortel, F. Blekman, E. S. Bols, J. D’Hondt, M. Delcourt, H. El Faham, S. Lowette, S. Moortgat, A. Morton, D. Müller, A. R. Sahasransu, S. Tavernier, W. Van Doninck, D. Beghin, B. Bilin, B. Clerbaux, G. De Lentdecker, L. Favart, A. Grebenyuk, A. K. Kalsi, K. Lee, M. Mahdavikhorrami, I. Makarenko, L. Moureaux, L. Pétré, A. Popov, N. Postiau, E. Starling, L. Thomas, M. Vanden Bemden, C. Vander Velde, P. Vanlaer, T. Cornelis, D. Dobur, J. Knolle, L. Lambrecht, G. Mestdach, M. Niedziela, C. Roskas, A. Samalan, K. Skovpen, M. Tytgat, B. Vermassen, L. Wezenbeek, A. Benecke, A. Bethani, G. Bruno, F. Bury, C. Caputo, P. David, C. Delaere, I. S. Donertas, A. Giammanco, K. Jaffel, Sa. Jain, V. Lemaitre, K. Mondal, J. Prisciandaro, A. Taliercio, M. Teklishyn, T. T. Tran, P. Vischia, S. Wertz, G. A. Alves, C. Hensel, A. Moraes, P. Rebello Teles, W. L. Aldá Júnior, M. Alves Gallo Pereira, M. Barroso Ferreira Filho, H. Brandao Malbouisson, W. Carvalho, J. Chinellato, E. M. Da Costa, G. G. Da Silveira, D. De Jesus Damiao, S. Fonseca De Souza, C. Mora Herrera, K. Mota Amarilo, L. Mundim, H. Nogima, A. Santoro, S. M. Silva Do Amaral, A. Sznajder, M. Thiel, F. Torres Da Silva De Araujo, A. Vilela Pereira, C. A. Bernardes, L. Calligaris, T. R. Fernandez Perez Tomei, E. M. Gregores, D. S. Lemos, P. G. Mercadante, S. F. Novaes, Sandra S. Padula, A. Aleksandrov, G. Antchev, R. Hadjiiska, P. Iaydjiev, M. Misheva, M. Rodozov, M. Shopova, G. Sultanov, A. Dimitrov, T. Ivanov, L. Litov, B. Pavlov, P. Petkov, A. Petrov, T. Cheng, T. Javaid, M. Mittal, L. Yuan, M. Ahmad, G. Bauer, C. Dozen, Z. Hu, J. Martins, Y. Wang, K. Yi, E. Chapon, G. M. Chen, H. S. Chen, M. Chen, F. Iemmi, A. Kapoor, D. Leggat, H. Liao, Z. -A. Liu, V. Milosevic, F. Monti, R. Sharma, J. Tao, J. Thomas-Wilsker, J. Wang, H. Zhang, J. Zhao, A. Agapitos, Y. An, Y. Ban, C. Chen, A. Levin, Q. Li, X. Lyu, Y. Mao, S. J. Qian, D. Wang, J. Xiao, H. Yang, M. Lu, Z. You, X. Gao, H. Okawa, Y. Zhang, Z. Lin, M. Xiao, C. Avila, A. Cabrera, C. Florez, J. Fraga, J. Mejia Guisao, F. Ramirez, J. D. Ruiz Alvarez, C. A. Salazar González, D. Giljanovic, N. Godinovic, D. Lelas, I. Puljak, Z. Antunovic, M. Kovac, T. Sculac, V. Brigljevic, D. Ferencek, D. Majumder, M. Roguljic, A. Starodumov, T. Susa, A. Attikis, K. Christoforou, E. Erodotou, A. Ioannou, G. Kole, M. Kolosova, S. Konstantinou, J. Mousa, C. Nicolaou, F. Ptochos, P. A. Razis, H. Rykaczewski, H. Saka, M. Finger, M. Finger, A. Kveton, E. Ayala, E. Carrera Jarrin, A. A. Abdelalim, E. Salama, M. A. Mahmoud, Y. Mohammed, S. Bhowmik, R. K. Dewanjee, K. Ehataht, M. Kadastik, S. Nandan, C. Nielsen, J. Pata, M. Raidal, L. Tani, C. Veelken, P. Eerola, L. Forthomme, H. Kirschenmann, K. Osterberg, M. Voutilainen, S. Bharthuar, E. Brücken, F. Garcia, J. Havukainen, M. S. Kim, R. Kinnunen, T. Lampén, K. Lassila-Perini, S. Lehti, T. Lindén, M. Lotti, L. Martikainen, M. Myllymäki, J. Ott, H. Siikonen, E. Tuominen, J. Tuominiemi, P. Luukka, H. Petrow, T. Tuuva, C. Amendola, M. Besancon, F. Couderc, M. Dejardin, D. Denegri, J. L. Faure, F. Ferri, S. Ganjour, P. Gras, G. Hamel de Monchenault, P. Jarry, B. Lenzi, E. Locci, J. Malcles, J. Rander, A. Rosowsky, M. Ö. Sahin, A. Savoy-Navarro, M. Titov, G. B. Yu, S. Ahuja, F. Beaudette, M. Bonanomi, A. Buchot Perraguin, P. Busson, A. Cappati, C. Charlot, O. Davignon, B. Diab, G. Falmagne, S. Ghosh, R. Granier de Cassagnac, A. Hakimi, I. Kucher, J. Motta, M. Nguyen, C. Ochando, P. Paganini, J. Rembser, R. Salerno, U. Sarkar, J. B. Sauvan, Y. Sirois, A. Tarabini, A. Zabi, A. Zghiche, J. -L. Agram, J. Andrea, D. Apparu, D. Bloch, G. Bourgatte, J. -M. Brom, E. C. Chabert, C. Collard, D. Darej, J. -C. Fontaine, U. Goerlach, C. Grimault, A. -C. Le Bihan, E. Nibigira, P. Van Hove, E. Asilar, S. Beauceron, C. Bernet, G. Boudoul, C. Camen, A. Carle, N. Chanon, D. Contardo, P. Depasse, H. El Mamouni, J. Fay, S. Gascon, M. Gouzevitch, B. Ille, I. B. Laktineh, H. Lattaud, A. Lesauvage, M. Lethuillier, L. Mirabito, S. Perries, K. Shchablo, V. Sordini, L. Torterotot, G. Touquet, M. Vander Donckt, S. Viret, A. Khvedelidze, I. Lomidze, Z. Tsamalaidze, V. Botta, L. Feld, K. Klein, M. Lipinski, D. Meuser, A. Pauls, N. Röwert, J. Schulz, M. Teroerde, A. Dodonova, D. Eliseev, M. Erdmann, P. Fackeldey, B. Fischer, S. Ghosh, T. Hebbeker, K. Hoepfner, F. Ivone, L. Mastrolorenzo, M. Merschmeyer, A. Meyer, G. Mocellin, S. Mondal, S. Mukherjee, D. Noll, A. Novak, T. Pook, A. Pozdnyakov, Y. Rath, H. Reithler, J. Roemer, A. Schmidt, S. C. Schuler, A. Sharma, L. Vigilante, S. Wiedenbeck, S. Zaleski, C. Dziwok, G. Flügge, W. Haj Ahmad, O. Hlushchenko, T. Kress, A. Nowack, C. Pistone, O. Pooth, D. Roy, A. Stahl, T. Ziemons, A. Zotz, H. Aarup Petersen, M. Aldaya Martin, P. Asmuss, S. Baxter, M. Bayatmakou, O. Behnke, A. Bermúdez Martínez, S. Bhattacharya, A. A. Bin Anuar, K. Borras, D. Brunner, A. Campbell, A. Cardini, C. Cheng, F. Colombina, S. Consuegra Rodríguez, G. Correia Silva, V. Danilov, M. De Silva, L. Didukh, G. Eckerlin, D. Eckstein, L. I. Estevez Banos, O. Filatov, E. Gallo, A. Geiser, A. Giraldi, A. Grohsjean, M. Guthoff, A. Jafari, N. Z. Jomhari, A. Kasem, M. Kasemann, H. Kaveh, C. Kleinwort, R. Kogler, D. Krücker, W. Lange, J. Lidrych, K. Lipka, W. Lohmann, R. Mankel, I. -A. Melzer-Pellmann, M. Mendizabal Morentin, J. Metwally, A. B. Meyer, M. Meyer, J. Mnich, A. Mussgiller, Y. Otarid, D. Pérez Adán, D. Pitzl, A. Raspereza, B. Ribeiro Lopes, J. Rübenach, A. Saggio, A. Saibel, M. Savitskyi, M. Scham, V. Scheurer, S. Schnake, P. Schütze, C. Schwanenberger, M. Shchedrolosiev, R. E. Sosa Ricardo, D. Stafford, N. Tonon, M. Van De Klundert, R. Walsh, D. Walter, Q. Wang, Y. Wen, K. Wichmann, L. Wiens, C. Wissing, S. Wuchterl, R. Aggleton, S. Albrecht, S. Bein, L. Benato, P. Connor, K. De Leo, M. Eich, F. Feindt, A. Fröhlich, C. Garbers, E. Garutti, P. Gunnellini, M. Hajheidari, J. Haller, A. Hinzmann, G. Kasieczka, R. Klanner, T. Kramer, V. Kutzner, J. Lange, T. Lange, A. Lobanov, A. Malara, A. Nigamova, K. J. Pena Rodriguez, M. Rieger, O. Rieger, P. Schleper, M. Schröder, J. Schwandt, J. Sonneveld, H. Stadie, G. Steinbrück, A. Tews, I. Zoi, J. Bechtel, S. Brommer, M. Burkart, E. Butz, R. Caspart, T. Chwalek, W. De Boer, A. Dierlamm, A. Droll, K. El Morabit, N. Faltermann, M. Giffels, J. o. Gosewisch, A. Gottmann, F. Hartmann, C. Heidecker, U. Husemann, P. Keicher, R. Koppenhöfer, S. Maier, M. Metzler, S. Mitra, Th. Müller, M. Neukum, A. Nürnberg, G. Quast, K. Rabbertz, J. Rauser, D. Savoiu, M. Schnepf, D. Seith, I. Shvetsov, H. J. Simonis, R. Ulrich, J. Van Der Linden, R. F. Von Cube, M. Wassmer, M. Weber, S. Wieland, R. Wolf, S. Wozniewski, S. Wunsch, G. Anagnostou, G. Daskalakis, T. Geralis, A. Kyriakis, A. Stakia, M. Diamantopoulou, D. Karasavvas, G. Karathanasis, P. Kontaxakis, C. K. Koraka, A. Manousakis-Katsikakis, A. Panagiotou, I. Papavergou, N. Saoulidou, K. Theofilatos, E. Tziaferi, K. Vellidis, E. Vourliotis, G. Bakas, K. Kousouris, I. Papakrivopoulos, G. Tsipolitis, A. Zacharopoulou, K. Adamidis, I. Bestintzanos, I. Evangelou, C. Foudas, P. Gianneios, P. Katsoulis, P. Kokkas, N. Manthos, I. Papadopoulos, J. Strologas, M. Csanád, K. Farkas, M. M. A. Gadallah, S. Lökös, P. Major, K. Mandal, A. Mehta, G. Pásztor, A. J. Rádl, O. Surányi, G. I. Veres, M. Bartók, G. Bencze, C. Hajdu, D. Horvath, F. Sikler, V. Veszpremi, S. Czellar, D. Fasanella, J. Karancsi, J. Molnar, Z. Szillasi, D. Teyssier, P. Raics, Z. L. Trocsanyi, B. Ujvari, T. Csorgo, F. Nemes, T. Novak, S. Choudhury, J. R. Komaragiri, D. Kumar, L. Panwar, P. C. Tiwari, S. Bansal, S. B. Beri, V. Bhatnagar, G. Chaudhary, S. Chauhan, N. Dhingra, R. Gupta, A. Kaur, M. Kaur, S. Kaur, P. Kumari, M. Meena, K. Sandeep, J. B. Singh, A. K. Virdi, A. Ahmed, A. Bhardwaj, B. C. Choudhary, M. Gola, S. Keshri, A. Kumar, M. Naimuddin, P. Priyanka, K. Ranjan, A. Shah, M. Bharti, R. Bhattacharya, S. Bhattacharya, D. Bhowmik, S. Dutta, S. Dutta, B. Gomber, M. Maity, P. Palit, P. K. Rout, G. Saha, B. Sahu, S. Sarkar, M. Sharan, B. Singh, S. Thakur, P. K. Behera, S. C. Behera, P. Kalbhor, A. Muhammad, R. Pradhan, P. R. Pujahari, A. Sharma, A. K. Sikdar, D. Dutta, V. Jha, V. Kumar, D. K. Mishra, K. Naskar, P. K. Netrakanti, L. M. Pant, P. Shukla, T. Aziz, S. Dugad, M. Kumar, G. B. Mohanty, S. Banerjee, R. Chudasama, M. Guchait, S. Karmakar, S. Kumar, G. Majumder, K. Mazumdar, S. Mukherjee, S. Bahinipati, C. Kar, P. Mal, T. Mishra, V. K. Muraleedharan Nair Bindhu, A. Nayak, P. Saha, N. Sur, S. K. Swain, D. Vats, K. Alpana, S. Dube, B. Kansal, A. Laha, S. Pandey, A. Rane, A. Rastogi, S. Sharma, H. Bakhshiansohi, E. Khazaie, M. Zeinali, S. Chenarani, S. M. Etesami, M. Khakzad, M. Mohammadi Najafabadi, M. Grunewald, M. Abbrescia, R. Aly, C. Aruta, A. Colaleo, D. Creanza, N. De Filippis, M. De Palma, A. Di Florio, A. Di Pilato, W. Elmetenawee, L. Fiore, A. Gelmi, M. Gul, G. Iaselli, M. Ince, S. Lezki, G. Maggi, M. Maggi, I. Margjeka, V. Mastrapasqua, S. My, S. Nuzzo, A. Pellecchia, A. Pompili, G. Pugliese, D. Ramos, A. Ranieri, G. Selvaggi, L. Silvestris, F. M. Simone, Ü. Sözbilir, R. Venditti, P. Verwilligen, G. Abbiendi, C. Battilana, D. Bonacorsi, L. Borgonovi, L. Brigliadori, R. Campanini, P. Capiluppi, A. Castro, F. R. Cavallo, M. Cuffiani, G. M. Dallavalle, T. Diotalevi, F. Fabbri, A. Fanfani, P. Giacomelli, L. Giommi, C. Grandi, L. Guiducci, S. Lo Meo, L. Lunerti, S. Marcellini, G. Masetti, F. L. Navarria, A. Perrotta, F. Primavera, A. M. Rossi, T. Rovelli, G. P. Siroli, S. Albergo, S. Costa, A. Di Mattia, R. Potenza, A. Tricomi, C. Tuve, G. Barbagli, A. Cassese, R. Ceccarelli, V. Ciulli, C. Civinini, R. D’Alessandro, E. Focardi, G. Latino, P. Lenzi, M. Lizzo, M. Meschini, S. Paoletti, R. Seidita, G. Sguazzoni, L. Viliani, L. Benussi, S. Bianco, D. Piccolo, M. Bozzo, F. Ferro, R. Mulargia, E. Robutti, S. Tosi, A. Benaglia, G. Boldrini, F. Brivio, F. Cetorelli, F. De Guio, M. E. Dinardo, P. Dini, S. Gennai, A. Ghezzi, P. Govoni, L. Guzzi, M. T. Lucchini, M. Malberti, S. Malvezzi, A. Massironi, D. Menasce, L. Moroni, M. Paganoni, D. Pedrini, B. S. Pinolini, S. Ragazzi, N. Redaelli, T. Tabarelli de Fatis, D. Valsecchi, D. Zuolo, S. Buontempo, F. Carnevali, N. Cavallo, A. De Iorio, F. Fabozzi, A. O. M. Iorio, L. Lista, S. Meola, P. Paolucci, B. Rossi, C. Sciacca, P. Azzi, N. Bacchetta, D. Bisello, P. Bortignon, A. Bragagnolo, R. Carlin, P. Checchia, T. Dorigo, U. Dosselli, F. Gasparini, U. Gasparini, G. Grosso, S. Y. Hoh, L. Layer, E. Lusiani, M. Margoni, A. T. Meneguzzo, J. Pazzini, P. Ronchese, R. Rossin, F. Simonetto, G. Strong, M. Tosi, H. Yarar, M. Zanetti, P. Zotto, A. Zucchetta, G. Zumerle, C. Aimè, A. Braghieri, S. Calzaferri, D. Fiorina, P. Montagna, S. P. Ratti, V. Re, C. Riccardi, P. Salvini, I. Vai, P. Vitulo, P. Asenov, G. M. Bilei, D. Ciangottini, L. Fanò, M. Magherini, G. Mantovani, V. Mariani, M. Menichelli, F. Moscatelli, A. Piccinelli, M. Presilla, A. Rossi, A. Santocchia, D. Spiga, T. Tedeschi, P. Azzurri, G. Bagliesi, V. Bertacchi, L. Bianchini, T. Boccali, E. Bossini, R. Castaldi, M. A. Ciocci, V. D’Amante, R. Dell’Orso, M. R. Di Domenico, S. Donato, A. Giassi, F. Ligabue, E. Manca, G. Mandorli, D. Matos Figueiredo, A. Messineo, F. Palla, S. Parolia, G. Ramirez-Sanchez, A. Rizzi, G. Rolandi, S. Roy Chowdhury, A. Scribano, N. Shafiei, P. Spagnolo, R. Tenchini, G. Tonelli, N. Turini, A. Venturi, P. G. Verdini, P. Barria, M. Campana, F. Cavallari, D. Del Re, E. Di Marco, M. Diemoz, E. Longo, P. Meridiani, G. Organtini, F. Pandolfi, R. Paramatti, C. Quaranta, S. Rahatlou, C. Rovelli, F. Santanastasio, L. Soffi, R. Tramontano, N. Amapane, R. Arcidiacono, S. Argiro, M. Arneodo, N. Bartosik, R. Bellan, A. Bellora, J. Berenguer Antequera, C. Biino, N. Cartiglia, S. Cometti, M. Costa, R. Covarelli, N. Demaria, B. Kiani, F. Legger, C. Mariotti, S. Maselli, E. Migliore, E. Monteil, M. Monteno, M. M. Obertino, G. Ortona, L. Pacher, N. Pastrone, M. Pelliccioni, G. L. Pinna Angioni, M. Ruspa, K. Shchelina, F. Siviero, V. Sola, A. Solano, D. Soldi, A. Staiano, M. Tornago, D. Trocino, A. Vagnerini, S. Belforte, V. Candelise, M. Casarsa, F. Cossutti, A. Da Rold, G. Della Ricca, G. Sorrentino, F. Vazzoler, S. Dogra, C. Huh, B. Kim, D. H. Kim, G. N. Kim, J. Kim, J. Lee, S. W. Lee, C. S. Moon, Y. D. Oh, S. I. Pak, B. C. Radburn-Smith, S. Sekmen, Y. C. Yang, H. Kim, D. H. Moon, B. Francois, T. J. Kim, J. Park, S. Cho, S. Choi, Y. Go, B. Hong, K. Lee, K. S. Lee, J. Lim, J. Park, S. K. Park, J. Yoo, J. Goh, A. Gurtu, H. S. Kim, Y. Kim, J. Almond, J. H. Bhyun, J. Choi, S. Jeon, J. Kim, J. S. Kim, S. Ko, H. Kwon, H. Lee, S. Lee, B. H. Oh, M. Oh, S. B. Oh, H. Seo, U. K. Yang, I. Yoon, W. Jang, D. Y. Kang, Y. Kang, S. Kim, B. Ko, J. S. H. Lee, Y. Lee, J. A. Merlin, I. C. Park, Y. Roh, M. S. Ryu, D. Song, I. J. Watson, S. Yang, S. Ha, H. D. Yoo, M. Choi, H. Lee, Y. Lee, I. Yu, T. Beyrouthy, Y. Maghrbi, K. Dreimanis, V. Veckalns, M. Ambrozas, A. Carvalho Antunes De Oliveira, A. Juodagalvis, A. Rinkevicius, G. Tamulaitis, N. Bin Norjoharuddeen, W. A. T. Wan Abdullah, M. N. Yusli, Z. Zolkapli, J. F. Benitez, A. Castaneda Hernandez, M. León Coello, J. A. Murillo Quijada, A. Sehrawat, L. Valencia Palomo, G. Ayala, H. Castilla-Valdez, E. De La Cruz-Burelo, I. Heredia-De La Cruz, R. Lopez-Fernandez, C. A. Mondragon Herrera, D. A. Perez Navarro, A. Sánchez Hernández, S. Carrillo Moreno, C. Oropeza Barrera, F. Vazquez Valencia, I. Pedraza, H. A. Salazar Ibarguen, C. Uribe Estrada, J. Mijuskovic, N. Raicevic, D. Krofcheck, P. H. Butler, A. Ahmad, M. I. Asghar, A. Awais, M. I. M. Awan, H. R. Hoorani, W. A. Khan, M. A. Shah, M. Shoaib, M. Waqas, V. Avati, L. Grzanka, M. Malawski, H. Bialkowska, M. Bluj, B. Boimska, M. Górski, M. Kazana, M. Szleper, P. Zalewski, K. Bunkowski, K. Doroba, A. Kalinowski, M. Konecki, J. Krolikowski, M. Araujo, P. Bargassa, D. Bastos, A. Boletti, P. Faccioli, M. Gallinaro, J. Hollar, N. Leonardo, T. Niknejad, M. Pisano, J. Seixas, O. Toldaiev, J. Varela, P. Adzic, M. Dordevic, P. Milenovic, J. Milosevic, M. Aguilar-Benitez, J. Alcaraz Maestre, A. Álvarez Fernández, I. Bachiller, M. Barrio Luna, Cristina F. Bedoya, C. A. Carrillo Montoya, M. Cepeda, M. Cerrada, N. Colino, B. De La Cruz, A. Delgado Peris, J. P. Fernández Ramos, J. Flix, M. C. Fouz, O. Gonzalez Lopez, S. Goy Lopez, J. M. Hernandez, M. I. Josa, J. León Holgado, D. Moran, Á. Navarro Tobar, C. Perez Dengra, A. Pérez-Calero Yzquierdo, J. Puerta Pelayo, I. Redondo, L. Romero, S. Sánchez Navas, L. Urda Gómez, C. Willmott, J. F. de Trocóniz, R. Reyes-Almanza, B. Alvarez Gonzalez, J. Cuevas, C. Erice, J. Fernandez Menendez, S. Folgueras, I. Gonzalez Caballero, J. R. González Fernández, E. Palencia Cortezon, C. Ramón Álvarez, V. Rodríguez Bouza, A. Soto Rodríguez, A. Trapote, N. Trevisani, C. Vico Villalba, J. A. Brochero Cifuentes, I. J. Cabrillo, A. Calderon, J. Duarte Campderros, M. Fernandez, C. Fernandez Madrazo, P. J. Fernández Manteca, A. García Alonso, G. Gomez, C. Martinez Rivero, P. Martinez Ruiz del Arbol, F. Matorras, P. Matorras Cuevas, J. Piedra Gomez, C. Prieels, T. Rodrigo, A. Ruiz-Jimeno, L. Scodellaro, I. Vila, J. M. Vizan Garcia, M. K. Jayananda, B. Kailasapathy, D. U. J. Sonnadara, D. D. C. Wickramarathna, W. G. D. Dharmaratna, K. Liyanage, N. Perera, N. Wickramage, T. K. Aarrestad, D. Abbaneo, J. Alimena, E. Auffray, G. Auzinger, J. Baechler, P. Baillon, D. Barney, J. Bendavid, M. Bianco, A. Bocci, T. Camporesi, M. Capeans Garrido, G. Cerminara, N. Chernyavskaya, S. S. Chhibra, M. Cipriani, L. Cristella, D. d’Enterria, A. Dabrowski, A. David, A. De Roeck, M. M. Defranchis, M. Deile, M. Dobson, M. Dünser, N. Dupont, A. Elliott-Peisert, N. Emriskova, F. Fallavollita, A. Florent, G. Franzoni, W. Funk, S. Giani, D. Gigi, K. Gill, F. Glege, L. Gouskos, M. Haranko, J. Hegeman, V. Innocente, T. James, P. Janot, J. Kaspar, J. Kieseler, M. Komm, N. Kratochwil, C. Lange, S. Laurila, P. Lecoq, A. Lintuluoto, K. Long, C. Lourenço, B. Maier, L. Malgeri, S. Mallios, M. Mannelli, A. C. Marini, F. Meijers, S. Mersi, E. Meschi, F. Moortgat, M. Mulders, S. Orfanelli, L. Orsini, F. Pantaleo, E. Perez, M. Peruzzi, A. Petrilli, G. Petrucciani, A. Pfeiffer, M. Pierini, D. Piparo, M. Pitt, H. Qu, T. Quast, D. Rabady, A. Racz, G. Reales Gutiérrez, M. Rovere, H. Sakulin, J. Salfeld-Nebgen, S. Scarfi, C. Schäfer, M. Selvaggi, A. Sharma, P. Silva, W. Snoeys, P. Sphicas, S. Summers, K. Tatar, V. R. Tavolaro, D. Treille, P. Tropea, A. Tsirou, G. P. Van Onsem, J. Wanczyk, K. A. Wozniak, W. D. Zeuner, L. Caminada, A. Ebrahimi, W. Erdmann, R. Horisberger, Q. Ingram, H. C. Kaestli, D. Kotlinski, M. Missiroli, L. Noehte, T. Rohe, K. Androsov, M. Backhaus, P. Berger, A. Calandri, A. De Cosa, G. Dissertori, M. Dittmar, M. Donegà, C. Dorfer, F. Eble, K. Gedia, F. Glessgen, T. A. Gómez Espinosa, C. Grab, D. Hits, W. Lustermann, A. -M. Lyon, R. A. Manzoni, L. Marchese, C. Martin Perez, M. T. Meinhard, F. Nessi-Tedaldi, J. Niedziela, F. Pauss, V. Perovic, S. Pigazzini, M. G. Ratti, M. Reichmann, C. Reissel, T. Reitenspiess, B. Ristic, D. Ruini, D. A. Sanz Becerra, V. Stampf, J. Steggemann, R. Wallny, D. H. Zhu, C. Amsler, P. Bärtschi, C. Botta, D. Brzhechko, M. F. Canelli, K. Cormier, A. De Wit, R. Del Burgo, J. K. Heikkilä, M. Huwiler, W. Jin, A. Jofrehei, B. Kilminster, S. Leontsinis, S. P. Liechti, A. Macchiolo, P. Meiring, V. M. Mikuni, U. Molinatti, I. Neutelings, A. Reimers, P. Robmann, S. Sanchez Cruz, K. Schweiger, M. Senger, Y. Takahashi, C. Adloff, C. M. Kuo, W. Lin, A. Roy, T. Sarkar, S. S. Yu, L. Ceard, Y. Chao, K. F. Chen, P. H. Chen, W. -S. Hou, Y. y. Li, R. -S. Lu, E. Paganis, A. Psallidas, A. Steen, H. y. Wu, E. Yazgan, P. r. Yu, B. Asavapibhop, C. Asawatangtrakuldee, N. Srimanobhas, F. Boran, S. Damarseckin, Z. S. Demiroglu, F. Dolek, I. Dumanoglu, E. Eskut, Y. Guler, E. Gurpinar Guler, C. Isik, O. Kara, A. Kayis Topaksu, U. Kiminsu, G. Onengut, K. Ozdemir, A. Polatoz, A. E. Simsek, B. Tali, U. G. Tok, S. Turkcapar, I. S. Zorbakir, B. Isildak, G. Karapinar, K. Ocalan, M. Yalvac, B. Akgun, I. O. Atakisi, E. Gülmez, M. Kaya, O. Kaya, Ö. Özçelik, S. Tekten, E. A. Yetkin, A. Cakir, K. Cankocak, Y. Komurcu, S. Sen, S. Cerci, I. Hos, B. Kaynak, S. Ozkorucuklu, H. Sert, D. Sunar Cerci, C. Zorbilmez, B. Grynyov, L. Levchuk, D. Anthony, E. Bhal, S. Bologna, J. J. Brooke, A. Bundock, E. Clement, D. Cussans, H. Flacher, J. Goldstein, G. P. Heath, H. F. Heath, L. Kreczko, B. Krikler, S. Paramesvaran, S. Seif El Nasr-Storey, V. J. Smith, N. Stylianou, K. Walkingshaw Pass, R. White, K. W. Bell, A. Belyaev, C. Brew, R. M. Brown, D. J. A. Cockerill, C. Cooke, K. V. Ellis, K. Harder, S. Harper, M. -L. Holmberg, J. Linacre, K. Manolopoulos, D. M. Newbold, E. Olaiya, D. Petyt, T. Reis, T. Schuh, C. H. Shepherd-Themistocleous, I. R. Tomalin, T. Williams, R. Bainbridge, P. Bloch, S. Bonomally, J. Borg, S. Breeze, O. Buchmuller, V. Cepaitis, G. S. Chahal, D. Colling, P. Dauncey, G. Davies, M. Della Negra, S. Fayer, G. Fedi, G. Hall, M. H. Hassanshahi, G. Iles, J. Langford, L. Lyons, A. -M. Magnan, S. Malik, A. Martelli, D. G. Monk, J. Nash, M. Pesaresi, D. M. Raymond, A. Richards, A. Rose, E. Scott, C. Seez, A. Shtipliyski, A. Tapper, K. Uchida, T. Virdee, M. Vojinovic, N. Wardle, S. N. Webb, D. Winterbottom, K. Coldham, J. E. Cole, A. Khan, P. Kyberd, I. D. Reid, L. Teodorescu, S. Zahid, S. Abdullin, A. Brinkerhoff, B. Caraway, J. Dittmann, K. Hatakeyama, A. R. Kanuganti, B. McMaster, N. Pastika, M. Saunders, S. Sawant, C. Sutantawibul, J. Wilson, R. Bartek, A. Dominguez, R. Uniyal, A. M. Vargas Hernandez, A. Buccilli, S. I. Cooper, D. Di Croce, S. V. Gleyzer, C. Henderson, C. U. Perez, P. Rumerio, C. West, A. Akpinar, A. Albert, D. Arcaro, C. Cosby, Z. Demiragli, E. Fontanesi, D. Gastler, S. May, J. Rohlf, K. Salyer, D. Sperka, D. Spitzbart, I. Suarez, A. Tsatsos, S. Yuan, D. Zou, G. Benelli, B. Burkle, X. Coubez, D. Cutts, M. Hadley, U. Heintz, J. M. Hogan, T. Kwon, G. Landsberg, K. T. Lau, D. Li, M. Lukasik, J. Luo, M. Narain, N. Pervan, S. Sagir, F. Simpson, E. Usai, W. Y. Wong, X. Yan, D. Yu, W. Zhang, J. Bonilla, C. Brainerd, R. Breedon, M. Calderon De La Barca Sanchez, M. Chertok, J. Conway, P. T. Cox, R. Erbacher, G. Haza, F. Jensen, O. Kukral, R. Lander, M. Mulhearn, D. Pellett, B. Regnery, D. Taylor, Y. Yao, F. Zhang, M. Bachtis, R. Cousins, A. Datta, D. Hamilton, J. Hauser, M. Ignatenko, M. A. Iqbal, T. Lam, W. A. Nash, S. Regnard, D. Saltzberg, B. Stone, V. Valuev, K. Burt, Y. Chen, R. Clare, J. W. Gary, M. Gordon, G. Hanson, G. Karapostoli, O. R. Long, N. Manganelli, M. Olmedo Negrete, W. Si, S. Wimpenny, Y. Zhang, J. G. Branson, P. Chang, S. Cittolin, S. Cooperstein, N. Deelen, D. Diaz, J. Duarte, R. Gerosa, L. Giannini, J. Guiang, R. Kansal, V. Krutelyov, R. Lee, J. Letts, M. Masciovecchio, F. Mokhtar, M. Pieri, B. V. Sathia Narayanan, V. Sharma, M. Tadel, A. Vartak, F. Würthwein, Y. Xiang, A. Yagil, N. Amin, C. Campagnari, M. Citron, A. Dorsett, V. Dutta, J. Incandela, M. Kilpatrick, J. Kim, B. Marsh, H. Mei, M. Oshiro, M. Quinnan, J. Richman, U. Sarica, F. Setti, J. Sheplock, P. Siddireddy, D. Stuart, S. Wang, A. Bornheim, O. Cerri, I. Dutta, J. M. Lawhorn, N. Lu, J. Mao, H. B. Newman, T. Q. Nguyen, M. Spiropulu, J. R. Vlimant, C. Wang, S. Xie, Z. Zhang, R. Y. Zhu, J. Alison, S. An, M. B. Andrews, P. Bryant, T. Ferguson, A. Harilal, C. Liu, T. Mudholkar, M. Paulini, A. Sanchez, W. Terrill, J. P. Cumalat, W. T. Ford, A. Hassani, E. MacDonald, R. Patel, A. Perloff, C. Savard, K. Stenson, K. A. Ulmer, S. R. Wagner, J. Alexander, S. Bright-Thonney, X. Chen, Y. Cheng, D. J. Cranshaw, S. Hogan, J. Monroy, J. R. Patterson, D. Quach, J. Reichert, M. Reid, A. Ryd, W. Sun, J. Thom, P. Wittich, R. Zou, M. Albrow, M. Alyari, G. Apollinari, A. Apresyan, A. Apyan, S. Banerjee, L. A. T. Bauerdick, D. Berry, J. Berryhill, P. C. Bhat, K. Burkett, J. N. Butler, A. Canepa, G. B. Cerati, H. W. K. Cheung, F. Chlebana, K. F. Di Petrillo, V. D. Elvira, Y. Feng, J. Freeman, Z. Gecse, L. Gray, D. Green, S. Grünendahl, O. Gutsche, R. M. Harris, R. Heller, T. C. Herwig, J. Hirschauer, B. Jayatilaka, S. Jindariani, M. Johnson, U. Joshi, T. Klijnsma, B. Klima, K. H. M. Kwok, S. Lammel, D. Lincoln, R. Lipton, T. Liu, C. Madrid, K. Maeshima, C. Mantilla, D. Mason, P. McBride, P. Merkel, S. Mrenna, S. Nahn, J. Ngadiuba, V. O’Dell, V. Papadimitriou, K. Pedro, C. Pena, O. Prokofyev, F. Ravera, A. Reinsvold Hall, L. Ristori, E. Sexton-Kennedy, N. Smith, A. Soha, W. J. Spalding, L. Spiegel, J. Strait, L. Taylor, S. Tkaczyk, N. V. Tran, L. Uplegger, E. W. Vaandering, H. A. Weber, D. Acosta, P. Avery, D. Bourilkov, L. Cadamuro, V. Cherepanov, F. Errico, R. D. Field, D. Guerrero, B. M. Joshi, M. Kim, E. Koenig, J. Konigsberg, A. Korytov, K. H. Lo, K. Matchev, N. Menendez, G. Mitselmakher, A. Muthirakalayil Madhu, N. Rawal, D. Rosenzweig, S. Rosenzweig, J. Rotter, K. Shi, J. Sturdy, J. Wang, E. Yigitbasi, X. Zuo, T. Adams, A. Askew, R. Habibullah, V. Hagopian, K. F. Johnson, R. Khurana, T. Kolberg, G. Martinez, H. Prosper, C. Schiber, O. Viazlo, R. Yohay, J. Zhang, M. M. Baarmand, S. Butalla, T. Elkafrawy, M. Hohlmann, R. Kumar Verma, D. Noonan, M. Rahmani, F. Yumiceva, M. R. Adams, H. Becerril Gonzalez, R. Cavanaugh, S. Dittmer, O. Evdokimov, C. E. Gerber, D. A. Hangal, D. J. Hofman, A. H. Merrit, C. Mills, G. Oh, T. Roy, S. Rudrabhatla, M. B. Tonjes, N. Varelas, J. Viinikainen, X. Wang, Z. Wu, Z. Ye, M. Alhusseini, K. Dilsiz, L. Emediato, R. P. Gandrajula, O. K. Köseyan, J. -P. Merlo, A. Mestvirishvili, J. Nachtman, H. Ogul, Y. Onel, A. Penzo, C. Snyder, E. Tiras, O. Amram, B. Blumenfeld, L. Corcodilos, J. Davis, M. Eminizer, A. V. Gritsan, S. Kyriacou, P. Maksimovic, J. Roskes, M. Swartz, T.Á. Vámi, A. Abreu, J. Anguiano, C. Baldenegro Barrera, P. Baringer, A. Bean, A. Bylinkin, Z. Flowers, T. Isidori, S. Khalil, J. King, G. Krintiras, A. Kropivnitskaya, M. Lazarovits, C. Le Mahieu, C. Lindsey, J. Marquez, N. Minafra, M. Murray, M. Nickel, C. Rogan, C. Royon, R. Salvatico, S. Sanders, E. Schmitz, C. Smith, J. D. Tapia Takaki, Q. Wang, Z. Warner, J. Williams, G. Wilson, S. Duric, A. Ivanov, K. Kaadze, D. Kim, Y. Maravin, T. Mitchell, A. Modak, K. Nam, F. Rebassoo, D. Wright, E. Adams, A. Baden, O. Baron, A. Belloni, S. C. Eno, N. J. Hadley, S. Jabeen, R. G. Kellogg, T. Koeth, S. Lascio, A. C. Mignerey, S. Nabili, C. Palmer, M. Seidel, A. Skuja, L. Wang, K. Wong, D. Abercrombie, G. Andreassi, R. Bi, W. Busza, I. A. Cali, Y. Chen, M. D’Alfonso, J. Eysermans, C. Freer, G. Gomez-Ceballos, M. Goncharov, P. Harris, M. Hu, M. Klute, D. Kovalskyi, J. Krupa, Y. -J. Lee, C. Mironov, C. Paus, D. Rankin, C. Roland, G. Roland, Z. Shi, G. S. F. Stephans, J. Wang, Z. Wang, B. Wyslouch, R. M. Chatterjee, A. Evans, J. Hiltbrand, Sh. Jain, M. Krohn, Y. Kubota, J. Mans, M. Revering, R. Rusack, R. Saradhy, N. Schroeder, N. Strobbe, M. A. Wadud, K. Bloom, M. Bryson, S. Chauhan, D. R. Claes, C. Fangmeier, L. Finco, F. Golf, C. Joo, I. Kravchenko, M. Musich, I. Reed, J. E. Siado, G. R. Snow, W. Tabb, F. Yan, A. G. Zecchinelli, G. Agarwal, H. Bandyopadhyay, L. Hay, I. Iashvili, A. Kharchilava, C. McLean, D. Nguyen, J. Pekkanen, S. Rappoccio, A. Williams, G. Alverson, E. Barberis, Y. Haddad, A. Hortiangtham, J. Li, G. Madigan, B. Marzocchi, D. M. Morse, V. Nguyen, T. Orimoto, A. Parker, L. Skinnari, A. Tishelman-Charny, T. Wamorkar, B. Wang, A. Wisecarver, D. Wood, S. Bhattacharya, J. Bueghly, Z. Chen, A. Gilbert, T. Gunter, K. A. Hahn, Y. Liu, N. Odell, M. H. Schmitt, M. Velasco, R. Band, R. Bucci, M. Cremonesi, A. Das, N. Dev, R. Goldouzian, M. Hildreth, K. Hurtado Anampa, C. Jessop, K. Lannon, J. Lawrence, N. Loukas, L. Lutton, J. Mariano, N. Marinelli, I. Mcalister, T. McCauley, C. Mcgrady, K. Mohrman, C. Moore, Y. Musienko, R. Ruchti, A. Townsend, M. Wayne, A. Wightman, M. Zarucki, L. Zygala, B. Bylsma, B. Cardwell, L. S. Durkin, B. Francis, C. Hill, M. Nunez Ornelas, K. Wei, B. L. Winer, B. R. Yates, F. M. Addesa, B. Bonham, P. Das, G. Dezoort, P. Elmer, A. Frankenthal, B. Greenberg, N. Haubrich, S. Higginbotham, A. Kalogeropoulos, G. Kopp, S. Kwan, D. Lange, D. Marlow, K. Mei, I. Ojalvo, J. Olsen, D. Stickland, C. Tully, S. Malik, S. Norberg, A. S. Bakshi, V. E. Barnes, R. Chawla, S. Das, L. Gutay, M. Jones, A. W. Jung, S. Karmarkar, D. Kondratyev, M. Liu, G. Negro, N. Neumeister, G. Paspalaki, S. Piperov, A. Purohit, J. F. Schulte, M. Stojanovic, J. Thieman, F. Wang, R. Xiao, W. Xie, J. Dolen, N. Parashar, A. Baty, T. Carnahan, M. Decaro, S. Dildick, K. M. Ecklund, S. Freed, P. Gardner, F. J. M. Geurts, A. Kumar, W. Li, B. P. Padley, R. Redjimi, W. Shi, A. G. Stahl Leiton, S. Yang, L. Zhang, Y. Zhang, A. Bodek, P. de Barbaro, R. Demina, J. L. Dulemba, C. Fallon, T. Ferbel, M. Galanti, A. Garcia-Bellido, O. Hindrichs, A. Khukhunaishvili, E. Ranken, R. Taus, B. Chiarito, J. P. Chou, A. Gandrakota, Y. Gershtein, E. Halkiadakis, A. Hart, M. Heindl, O. Karacheban, I. Laflotte, A. Lath, R. Montalvo, K. Nash, M. Osherson, S. Salur, S. Schnetzer, S. Somalwar, R. Stone, S. A. Thayil, S. Thomas, H. Wang, H. Acharya, A. G. Delannoy, S. Fiorendi, S. Spanier, O. Bouhali, M. Dalchenko, A. Delgado, R. Eusebi, J. Gilmore, T. Huang, T. Kamon, H. Kim, S. Luo, S. Malhotra, R. Mueller, D. Overton, D. Rathjens, A. Safonov, N. Akchurin, J. Damgov, V. Hegde, S. Kunori, K. Lamichhane, S. W. Lee, T. Mengke, S. Muthumuni, T. Peltola, I. Volobouev, Z. Wang, A. Whitbeck, E. Appelt, S. Greene, A. Gurrola, W. Johns, A. Melo, H. Ni, K. Padeken, F. Romeo, P. Sheldon, S. Tuo, J. Velkovska, M. W. Arenton, B. Cox, G. Cummings, J. Hakala, R. Hirosky, M. Joyce, A. Ledovskoy, A. Li, C. Neu, C. E. Perez Lara, B. Tannenwald, S. White, E. Wolfe, N. Poudyal, K. Black, T. Bose, C. Caillol, S. Dasu, I. De Bruyn, P. Everaerts, F. Fienga, C. Galloni, H. He, M. Herndon, A. Hervé, U. Hussain, A. Lanaro, A. Loeliger, R. Loveless, J. Madhusudanan Sreekala, A. Mallampalli, A. Mohammadi, D. Pinna, A. Savin, V. Shang, V. Sharma, W. H. Smith, D. Teague, S. Trembath-Reichert, W. Vetens, S. Afanasiev, V. Andreev, Yu. Andreev, T. Aushev, M. Azarkin, A. Babaev, A. Belyaev, V. Blinov, E. Boos, V. Borshch, D. Budkouski, O. Bychkova, M. Chadeeva, V. Chekhovsky, A. Dermenev, T. Dimova, I. Dremin, M. Dubinin, L. Dudko, V. Epshteyn, A. Ershov, G. Gavrilov, V. Gavrilov, S. Gninenko, V. Golovtcov, N. Golubev, I. Golutvin, I. Gorbunov, A. Gribushin, V. Ivanchenko, Y. Ivanov, V. Kachanov, L. Kardapoltsev, V. Karjavine, A. Karneyeu, V. Kim, M. Kirakosyan, D. Kirpichnikov, M. Kirsanov, V. Klyukhin, O. Kodolova, D. Konstantinov, V. Korenkov, A. Kozyrev, N. Krasnikov, E. Kuznetsova, A. Lanev, A. Litomin, N. Lychkovskaya, V. Makarenko, A. Malakhov, V. Matveev, V. Murzin, A. Nikitenko, S. Obraztsov, V. Okhotnikov, V. Oreshkin, A. Oskin, I. Ovtin, V. Palichik, P. Parygin, A. Pashenkov, V. Perelygin, S. Petrushanko, G. Pivovarov, V. Popov, E. Popova, O. Radchenko, V. Rusinov, M. Savina, V. Savrin, D. Seitova, V. Shalaev, S. Shmatov, S. Shulha, Y. Skovpen, S. Slabospitskii, I. Smirnov, V. Smirnov, A. Snigirev, D. Sosnov, A. Stepennov, V. Sulimov, E. Tcherniaev, A. Terkulov, O. Teryaev, M. Toms, A. Toropin, L. Uvarov, A. Uzunian, E. Vlasov, S. Volkov, A. Vorobyev, N. Voytishin, B. S. Yuldashev, A. Zarubin, I. Zhizhin, A. Zhokin, CMS Collaboration

**Affiliations:** 1 Yerevan Physics Institute, Yerevan, Armenia; 2 Institut für Hochenergiephysik, Vienna, Austria; 3 Universiteit Antwerpen, Antwerpen, Belgium; 4 Vrije Universiteit Brussel, Brussel, Belgium; 5 Université Libre de Bruxelles, Bruxelles, Belgium; 6 Ghent University, Ghent, Belgium; 7 Université Catholique de Louvain, Louvain-la-Neuve, Belgium; 8 Centro Brasileiro de Pesquisas Fisicas, Rio de Janeiro, Brazil; 9 Universidade do Estado do Rio de Janeiro, Rio de Janeiro, Brazil; 10 Universidade Estadual Paulista, Universidade Federal do ABC, São Paulo, Brazil; 11 Institute for Nuclear Research and Nuclear Energy, Bulgarian Academy of Sciences, Sofia, Bulgaria; 12 University of Sofia, Sofia, Bulgaria; 13 Beihang University, Beijing, China; 14 Department of Physics, Tsinghua University, Beijing, China; 15 Institute of High Energy Physics, Beijing, China; 16 State Key Laboratory of Nuclear Physics and Technology, Peking University, Beijing, China; 17 Sun Yat-Sen University, Guangzhou, China; 18 Institute of Modern Physics and Key Laboratory of Nuclear Physics and Ion-beam Application (MOE)-Fudan University, Shanghai, China; 19 Zhejiang University, Hangzhou, Zhejiang, China; 20 Universidad de Los Andes, Bogota, Colombia; 21 Universidad de Antioquia, Medellin, Colombia; 22 University of Split, Faculty of Electrical Engineering, Mechanical Engineering and Naval Architecture, Split, Croatia; 23 University of Split, Faculty of Science, Split, Croatia; 24 Institute Rudjer Boskovic, Zagreb, Croatia; 25 University of Cyprus, Nicosia, Cyprus; 26 Charles University, Prague, Czech Republic; 27 Escuela Politecnica Nacional, Quito, Ecuador; 28 Universidad San Francisco de Quito, Quito, Ecuador; 29 Academy of Scientific Research and Technology of the Arab Republic of Egypt, Egyptian Network of High Energy Physics, Cairo, Egypt; 30 Center for High Energy Physics (CHEP-FU), Fayoum University, El-Fayoum, Egypt; 31 National Institute of Chemical Physics and Biophysics, Tallinn, Estonia; 32 Department of Physics, University of Helsinki, Helsinki, Finland; 33 Helsinki Institute of Physics, Helsinki, Finland; 34 Lappeenranta-Lahti University of Technology, Lappeenranta, Finland; 35 IRFU, CEA, Université Paris-Saclay, Gif-sur-Yvette, France; 36 Laboratoire Leprince-Ringuet, CNRS/IN2P3, Ecole Polytechnique, Institut Polytechnique de Paris, Palaiseau, France; 37 Université de Strasbourg, CNRS, IPHC UMR 7178, Strasbourg, France; 38 Institut de Physique des 2 Infinis de Lyon (IP2I ), Villeurbanne, France; 39 Georgian Technical University, Tbilisi, Georgia; 40 RWTH Aachen University, I. Physikalisches Institut, Aachen, Germany; 41 RWTH Aachen University, III. Physikalisches Institut A, Aachen, Germany; 42 RWTH Aachen University, III. Physikalisches Institut B, Aachen, Germany; 43 Deutsches Elektronen-Synchrotron, Hamburg, Germany; 44 University of Hamburg, Hamburg, Germany; 45 Karlsruher Institut fuer Technologie, Karlsruhe, Germany; 46 Institute of Nuclear and Particle Physics (INPP), NCSR Demokritos, Aghia Paraskevi, Greece; 47 National and Kapodistrian University of Athens, Athens, Greece; 48 National Technical University of Athens, Athens, Greece; 49 University of Ioánnina, Ioánnina, Greece; 50 MTA-ELTE Lendület CMS Particle and Nuclear Physics Group, Eötvös Loránd University, Budapest, Hungary; 51 Wigner Research Centre for Physics, Budapest, Hungary; 52 Institute of Nuclear Research ATOMKI, Debrecen, Hungary; 53 Institute of Physics, University of Debrecen, Debrecen, Hungary; 54 Karoly Robert Campus, MATE Institute of Technology, Gyongyos, Hungary; 55 Indian Institute of Science (IISc), Bangalore, India; 56 Panjab University, Chandigarh, India; 57 University of Delhi, Delhi, India; 58 Saha Institute of Nuclear Physics, HBNI, Kolkata, India; 59 Indian Institute of Technology Madras, Madras, India; 60 Bhabha Atomic Research Centre, Mumbai, India; 61 Tata Institute of Fundamental Research-A, Mumbai, India; 62 Tata Institute of Fundamental Research-B, Mumbai, India; 63 National Institute of Science Education and Research, An OCC of Homi Bhabha National Institute, Bhubaneswar, Odisha India; 64 Indian Institute of Science Education and Research (IISER), Pune, India; 65 Isfahan University of Technology, Isfahan, Iran; 66 Institute for Research in Fundamental Sciences (IPM), Tehran, Iran; 67 University College Dublin, Dublin, Ireland; 68 INFN Sezione di Bari, Università di Bari, Politecnico di Bari, Bari, Italy; 69 INFN Sezione di Bologna, Università di Bologna, Bologna, Italy; 70 INFN Sezione di Catania, Università di Catania, Catania, Italy; 71 INFN Sezione di Firenze, Università di Firenze, Florence, Italy; 72 INFN Laboratori Nazionali di Frascati, Frascati, Italy; 73 INFN Sezione di Genova, Università di Genova, Genoa, Italy; 74 INFN Sezione di Milano-Bicocca, Università di Milano-Bicocca, Milan, Italy; 75 INFN Sezione di Napoli, Università di Napoli ’Federico II’, Napoli, Italy; Università della Basilicata, Potenza, Italy; Università G. Marconi, Rome, Italy; 76 INFN Sezione di Padova, Università di Padova, Padua, Italy; Università di Trento, Trento, Italy; 77 INFN Sezione di Pavia, Università di Pavia, Pavia, Italy; 78 INFN Sezione di Perugia, Università di Perugia, Perugia, Italy; 79 INFN Sezione di Pisa, Università di Pisa, Scuola Normale Superiore di Pisa, Pisa, Italy; Università di Siena, Siena, Italy; 80 INFN Sezione di Roma, Sapienza Università di Roma, Rome, Italy; 81 INFN Sezione di Torino, Università di Torino, Turin, Italy; Università del Piemonte Orientale, Novara, Italy; 82 INFN Sezione di Trieste, Università di Trieste, Trieste, Italy; 83 Kyungpook National University, Daegu, Korea; 84 Chonnam National University, Institute for Universe and Elementary Particles, Kwangju, Korea; 85 Hanyang University, Seoul, Korea; 86 Korea University, Seoul, Korea; 87 Department of Physics, Kyung Hee University, Seoul, Korea; 88 Sejong University, Seoul, Korea; 89 Seoul National University, Seoul, Korea; 90 University of Seoul, Seoul, Korea; 91 Department of Physics, Yonsei University, Seoul, Korea; 92 Sungkyunkwan University, Suwon, Korea; 93 College of Engineering and Technology, American University of the Middle East (AUM), Dasman, Kuwait; 94 Riga Technical University, Riga, Latvia; 95 Vilnius University, Vilnius, Lithuania; 96 National Centre for Particle Physics, Universiti Malaya, Kuala Lumpur, Malaysia; 97 Universidad de Sonora (UNISON), Hermosillo, Mexico; 98 Centro de Investigacion y de Estudios Avanzados del IPN, Mexico City, Mexico; 99 Universidad Iberoamericana, Mexico City, Mexico; 100 Benemerita Universidad Autonoma de Puebla, Puebla, Mexico; 101 University of Montenegro, Podgorica, Montenegro; 102 University of Auckland, Auckland, New Zealand; 103 University of Canterbury, Christchurch, New Zealand; 104 National Centre for Physics, Quaid-i-Azam University, Islamabad, Pakistan; 105 AGH University of Science and Technology Faculty of Computer Science, Electronics and Telecommunications, Krakow, Poland; 106 National Centre for Nuclear Research, Swierk, Poland; 107 Institute of Experimental Physics, Faculty of Physics, University of Warsaw, Warsaw, Poland; 108 Laboratório de Instrumentação e Física Experimental de Partículas, Lisbon, Portugal; 109 VINCA Institute of Nuclear Sciences, University of Belgrade, Belgrade, Serbia; 110 Centro de Investigaciones Energéticas Medioambientales y Tecnológicas (CIEMAT), Madrid, Spain; 111 Universidad Autónoma de Madrid, Madrid, Spain; 112 Universidad de Oviedo, Instituto Universitario de Ciencias y Tecnologías Espaciales de Asturias (ICTEA), Oviedo, Spain; 113 Instituto de Física de Cantabria (IFCA), CSIC-Universidad de Cantabria, Santander, Spain; 114 University of Colombo, Colombo, Sri Lanka; 115 Department of Physics, University of Ruhuna, Matara, Sri Lanka; 116 CERN, European Organization for Nuclear Research, Geneva, Switzerland; 117 Paul Scherrer Institut, Villigen, Switzerland; 118 ETH Zurich-Institute for Particle Physics and Astrophysics (IPA), Zurich, Switzerland; 119 Universität Zürich, Zurich, Switzerland; 120 National Central University, Chung-Li, Taiwan; 121 National Taiwan University (NTU), Taipei, Taiwan; 122 Department of Physics, Chulalongkorn University, Faculty of Science, Bangkok, Thailand; 123 Physics Department, Science and Art Faculty, Çukurova University, Adana, Turkey; 124 Physics Department, Middle East Technical University, Ankara, Turkey; 125 Bogazici University, Istanbul, Turkey; 126 Istanbul Technical University, Istanbul, Turkey; 127 Istanbul University, Istanbul, Turkey; 128 Institute for Scintillation Materials of National Academy of Science of Ukraine, Kharkiv, Ukraine; 129 National Science Centre, Kharkiv Institute of Physics and Technology, Kharkiv, Ukraine; 130 University of Bristol, Bristol, UK; 131 Rutherford Appleton Laboratory, Didcot, UK; 132 Imperial College, London, UK; 133 Brunel University, Uxbridge, UK; 134 Baylor University, Waco, TX USA; 135 Catholic University of America, Washington, DC USA; 136 The University of Alabama, Tuscaloosa, AL USA; 137 Boston University, Boston, MA USA; 138 Brown University, Providence, RI USA; 139 University of California, Davis, Davis, CA USA; 140 University of California, Los Angeles, CA USA; 141 University of California, Riverside, Riverside, CA USA; 142 University of California, San Diego, La Jolla, CA USA; 143 Department of Physics, University of California, Santa Barbara, Santa Barbara, CA USA; 144 California Institute of Technology, Pasadena, CA USA; 145 Carnegie Mellon University, Pittsburgh, PA USA; 146 University of Colorado Boulder, Boulder, CO USA; 147 Cornell University, Ithaca, NY USA; 148 Fermi National Accelerator Laboratory, Batavia, IL USA; 149 University of Florida, Gainesville, FL USA; 150 Florida State University, Tallahassee, FL USA; 151 Florida Institute of Technology, Melbourne, FL USA; 152 University of Illinois at Chicago (UIC), Chicago, IL USA; 153 The University of Iowa, Iowa City, IA USA; 154 Johns Hopkins University, Baltimore, MD USA; 155 The University of Kansas, Lawrence, KS USA; 156 Kansas State University, Manhattan, KS USA; 157 Lawrence Livermore National Laboratory, Livermore, CA USA; 158 University of Maryland, College Park, MD USA; 159 Massachusetts Institute of Technology, Cambridge, MA USA; 160 University of Minnesota, Minneapolis, MN USA; 161 University of Nebraska-Lincoln, Lincoln, NE USA; 162 State University of New York at Buffalo, Buffalo, NY USA; 163 Northeastern University, Boston, MA USA; 164 Northwestern University, Evanston, IL USA; 165 University of Notre Dame, Notre Dame, IN USA; 166 The Ohio State University, Columbus, OH USA; 167 Princeton University, Princeton, NJ USA; 168 University of Puerto Rico, Mayaguez, PR USA; 169 Purdue University, West Lafayette, IN USA; 170 Purdue University Northwest, Hammond, IN USA; 171 Rice University, Houston, TX USA; 172 University of Rochester, Rochester, NY USA; 173 Rutgers, The State University of New Jersey, Piscataway, NJ USA; 174 University of Tennessee, Knoxville, TN USA; 175 Texas A &M University, College Station, TX USA; 176 Texas Tech University, Lubbock, TX USA; 177 Vanderbilt University, Nashville, TN USA; 178 University of Virginia, Charlottesville, VA USA; 179 Wayne State University, Detroit, MI USA; 180 University of Wisconsin-Madison, Madison, WI USA; 181 Authors Affiliated with an Institute or an International Laboratory Covered by a Cooperation Agreement with CERN, Geneva, Switzerland; 182 Yerevan State University, Yerevan, Armenia; 183 TU Wien, Vienna, Austria; 184 Institute of Basic and Applied Sciences, Faculty of Engineering, Arab Academy for Science, Technology and Maritime Transport, Alexandria, Egypt; 185 Université Libre de Bruxelles, Bruxelles, Belgium; 186 Universidade Estadual de Campinas, Campinas, Brazil; 187 Federal University of Rio Grande do Sul, Porto Alegre, Brazil; 188 The University of the State of Amazonas, Manaus, Brazil; 189 University of Chinese Academy of Sciences, Beijing, China; 190 UFMS, Nova Andradina, Brazil; 191 Nanjing Normal University Department of Physics, Nanjing, China; 192 The University of Iowa, Iowa City, IA USA; 193 University of Chinese Academy of Sciences, Beijing, China; 194 an institute or an international laboratory covered by a cooperation agreement with CERN, Geneva, Switzerland; 195 Helwan University, Cairo, Egypt; 196 Zewail City of Science and Technology, Zewail, Egypt; 197 British University in Egypt, Cairo, Egypt; 198 Ain Shams University, Cairo, Egypt; 199 Purdue University, West Lafayette, IN USA; 200 Université de Haute Alsace, Mulhouse, France; 201 Erzincan Binali Yildirim University, Erzincan, Turkey; 202 CERN, European Organization for Nuclear Research, Geneva, Switzerland; 203 RWTH Aachen University, III. Physikalisches Institut A, Aachen, Germany; 204 University of Hamburg, Hamburg, Germany; 205 Isfahan University of Technology, Isfahan, Iran; 206 Brandenburg University of Technology, Cottbus, Germany; 207 Forschungszentrum Jülich, Juelich, Germany; 208 Physics Department, Faculty of Science, Assiut University, Assiut, Egypt; 209 Karoly Robert Campus, MATE Institute of Technology, Gyongyos, Hungary; 210 Institute of Physics, University of Debrecen, Debrecen, Hungary; 211 Institute of Nuclear Research ATOMKI, Debrecen, Hungary; 212 MTA-ELTE Lendület CMS Particle and Nuclear Physics Group, Eötvös Loránd University, Budapest, Hungary; 213 Wigner Research Centre for Physics, Budapest, Hungary; 214 Punjab Agricultural University, Ludhiana, India; 215 Shoolini University, Solan, India; 216 University of Hyderabad, Hyderabad, India; 217 University of Visva-Bharati, Santiniketan, India; 218 Indian Institute of Technology (IIT), Mumbai, India; 219 IIT Bhubaneswar, Bhubaneswar, India; 220 Institute of Physics, Bhubaneswar, India; 221 Deutsches Elektronen-Synchrotron, Hamburg, Germany; 222 Sharif University of Technology, Tehran, Iran; 223 Department of Physics, University of Science and Technology of Mazandaran, Behshahr, Iran; 224 Italian National Agency for New Technologies, Energy and Sustainable Economic Development, Bologna, Italy; 225 Centro Siciliano di Fisica Nucleare e di Struttura Della Materia, Catania, Italy; 226 Scuola Superiore Meridionale, Università di Napoli ‘Federico II’, Napoli, Italy; 227 Università di Napoli ’Federico II’, Naples, Italy; 228 Consiglio Nazionale delle Ricerche-Istituto Officina dei Materiali, Perugia, Italy; 229 Consejo Nacional de Ciencia y Tecnología, Mexico City, Mexico; 230 IRFU, CEA, Université Paris-Saclay, Gif-sur-Yvette, France; 231 Faculty of Physics, University of Belgrade, Belgrade, Serbia; 232 Trincomalee Campus, Eastern University, Sri Lanka, Nilaveli, Sri Lanka; 233 INFN Sezione di Pavia, Università di Pavia, Pavia, Italy; 234 National and Kapodistrian University of Athens, Athens, Greece; 235 Ecole Polytechnique Fédérale Lausanne, Lausanne, Switzerland; 236 Universität Zürich, Zurich, Switzerland; 237 Stefan Meyer Institute for Subatomic Physics, Vienna, Austria; 238 Laboratoire d’Annecy-le-Vieux de Physique des Particules, IN2P3-CNRS, Annecy-le-Vieux, France; 239 Şırnak University, Sirnak, Turkey; 240 Near East University, Research Center of Experimental Health Science, Mersin, Turkey; 241 Konya Technical University, Konya, Turkey; 242 Izmir Bakircay University, Izmir, Turkey; 243 Adiyaman University, Adiyaman, Turkey; 244 Ozyegin University, Istanbul, Turkey; 245 Necmettin Erbakan University, Konya, Turkey; 246 Bozok Universitetesi Rektörlügü, Yozgat, Turkey; 247 Marmara University, Istanbul, Turkey; 248 Milli Savunma University, Istanbul, Turkey; 249 Kafkas University, Kars, Turkey; 250 Istanbul Bilgi University, Istanbul, Turkey; 251 Hacettepe University, Ankara, Turkey; 252 Istanbul University-Cerrahpasa, Faculty of Engineering, Istanbul, Turkey; 253 Vrije Universiteit Brussel, Brussels, Belgium; 254 School of Physics and Astronomy, University of Southampton, Southampton, UK; 255 IPPP Durham University, Durham, UK; 256 Monash University, Faculty of Science, Clayton, Australia; 257 Università di Torino, Turin, Italy; 258 Bethel University, St. Paul, MN USA; 259 Karamanoğlu Mehmetbey University, Karaman, Turkey; 260 California Institute of Technology, Pasadena, CA USA; 261 Bingol University, Bingol, Turkey; 262 Georgian Technical University, Tbilisi, Georgia; 263 Sinop University, Sinop, Turkey; 264 Erciyes University, Kayseri, Turkey; 265 Institute of Modern Physics and Key Laboratory of Nuclear Physics and Ion-beam Application (MOE)-Fudan University, Shanghai, China; 266 Texas A &M University at Qatar, Doha, Qatar; 267 Kyungpook National University, Daegu, Korea; 268 Another Institute or International Laboratory Covered by a Cooperation Agreement with CERN, Geneva, Switzerland; 269 Yerevan Physics Institute, Yerevan, Armenia; 270 University of Florida, Gainesville, FL USA; 271 Imperial College, London, UK; 272 Institute of Nuclear Physics of the Uzbekistan Academy of Sciences, Tashkent, Uzbekistan; 273 CERN, 1211 Geneva 23, Switzerland

## Abstract

Multijet events at large transverse momentum ($p_{\textrm{T}}$) are measured at $\sqrt{s}=13\,\text {TeV}$ using data recorded with the CMS detector at the LHC, corresponding to an integrated luminosity of $36.3{\,\text {fb}^{-1}}$. The multiplicity of jets with $p_{\textrm{T}} >50\,\text {GeV}$ that are produced in association with a high-$p_{\textrm{T}}$ dijet system is measured in various ranges of the $p_{\textrm{T}}$ of the jet with the highest transverse momentum and as a function of the azimuthal angle difference $\varDelta \phi _{1,2}$ between the two highest $p_{\textrm{T}}$ jets in the dijet system. The differential production cross sections are measured as a function of the transverse momenta of the four highest $p_{\textrm{T}}$ jets. The measurements are compared with leading and next-to-leading order matrix element calculations supplemented with simulations of parton shower, hadronization, and multiparton interactions. In addition, the measurements are compared with next-to-leading order matrix element calculations combined with transverse-momentum dependent parton densities and transverse-momentum dependent parton shower.

## Introduction

The production of jets, which are reconstructed from a stream of hadrons coming from the fragmentation of energetic partons, is described by the theory of strong interactions, quantum chromodynamics (QCD). In proton–proton ($\text {p}\text {p}$) collisions, at leading order (LO) in the strong coupling $\alpha _\textrm{S}$, two colliding partons from the incident protons scatter and produce two high transverse-momentum ($p_{\textrm{T}}$) partons in the final state. The jets that originate from such a process are strongly correlated in the transverse plane, and the azimuthal angle difference between them, $\varDelta \phi _{1,2}$, should be close to $\pi$. However, higher-order corrections to the lowest order process will result in a decorrelation in the azimuthal plane, and $\varDelta \phi _{1,2}$ will significantly deviate from $\pi$. These corrections can be due to either hard parton radiation, calculated at the matrix element (ME) level at next-to-leading order (NLO), or softer multiple parton radiation described by parton showers. In a recent approach [1], transverse-momentum dependent (TMD) parton densities are obtained with the parton-branching method [2, 3] (PB-TMDs). These PB-TMDs were combined with NLO ME calculations [4] supplemented with PB initial-state parton showers [5], leading to predictions where the initial-state parton shower is determined by the PB-TMD densities without tunable parameters. In Drell-Yan production at the LHC this approach leads to a good description of the measurements [6], whereas other approaches need specific tunes. Therefore, it is interesting to study the PB prediction in a jet environment, especially since the PB-TMD initial-state shower also becomes important. Although $\varDelta \phi _{1,2}$ is an inclusive observable, it is interesting for the theoretical understanding of the complete event to measure the multiplicity of jets in different regions of $\varDelta \phi _{1,2}$ and the transverse momenta of the first four jets. The $\varDelta \phi _{1,2}$ measurement is mainly sensitive to initial-state parton showers at an inclusive level, whereas the measurement of the jet multiplicity in different regions of $\varDelta \phi _{1,2}$ illustrates how many high-$p_{\textrm{T}}$ jets contribute to the $\varDelta \phi _{1,2}$ decorrelation.

The azimuthal correlation in high-$p_{\textrm{T}}$ dijet events was measured previously at: the Fermilab Tevatron in proton-antiproton collisions by the D0 Collaboration at $\sqrt{s}=1.96\,\text {TeV}$ [7, 8]; and at the CERN LHC in $\text {p}\text {p}$ collisions by both the ATLAS Collaboration at $\sqrt{s}=7\,\text {TeV}$ [9] and the CMS Collaboration at $\sqrt{s}=7$, 8, and $13\,\text {TeV}$ [10–13].

In this paper, we describe new measurements of dijet events with rapidity $|y | < 2.5$ and with transverse momenta of the leading jet $p_{\textrm{T1}} > 200\,\text {GeV}$ and the subleading jet $p_{\textrm{T2}} > 100\,\text {GeV}$. The multiplicity of jets with $p_{\textrm{T}} > 50\,\text {GeV}$ is measured in bins of $p_{\textrm{T1}}$ and $\varDelta \phi _{1,2}$. The jet multiplicity in bins of $\varDelta \phi _{1,2}$ provides information on the $\varDelta \phi _{1,2}$ decorrelation. The cross sections for the four leading jets are measured as a function of $p_{\textrm{T}}$ of each jet, which can give additional information on the structure of the higher-order corrections.

This paper is organized as follows; in Sect. 2, a brief summary of the CMS detector and the relevant components is given. In Sect. 3, the theoretical models for comparison at detector level, as well as with the final results are described. Section 4 gives an overview of the analysis, with the event selection, data correction, and a discussion of the uncertainties. The final results and comparison with theoretical predictions are discussed in Sect. 5.

## The CMS detector and event reconstruction

The central feature of the CMS apparatus is a superconducting solenoid of 6$\,\text {m}$ internal diameter, providing a magnetic field of 3.8$\,\text {T}$. Within the solenoid volume are silicon pixel and strip tracker detectors, a lead tungstate crystal electromagnetic calorimeter (ECAL), and a brass and scintillator hadron calorimeter (HCAL), each composed of a barrel part and two endcap sections.

Events of interest are selected using a two-tiered trigger system. The first level, composed of custom hardware processors, uses information from the calorimeters and muon detectors to select events at a rate of around 100$\,\text {kHz}$ within a fixed latency of about 4$\,\upmu \text {s}$  [14]. The second level, known as the high-level trigger (HLT), consists of a farm of processors running a version of the full event reconstruction software optimized for fast processing, while reduces the event rate to around 1$\,\text {kHz}$ for data storage [15].

During the 2016 data-taking period, a gradual shift in the timing of the inputs of the ECAL first level trigger in the pseudorapidity region $|\eta | > 2.0$, also known as “prefiring”, caused some trigger inefficiencies [14]. For events containing a jet with $p_{\textrm{T}} > 100\,\text {GeV}$, in the region $2.5< |\eta |< 3.0$ the efficiency loss is 10–20%, depending on $p_{\textrm{T}}$, $\eta$, and the data-taking period. Correction factors were computed from data and applied to the acceptance evaluated by simulation.

The particle-flow (PF) algorithm [16] reconstructs and identifies each individual particle in an event, with an optimized combination of information from the various elements of the CMS detector. The energy of charged hadrons is determined from a combination of their momentum measured in the tracker and the matching ECAL and HCAL energy deposits, corrected for the response function of the calorimeters to hadronic showers. The energy of neutral hadrons is obtained from the corresponding corrected ECAL and HCAL energies.

The candidate vertex with the largest value of summed physics-object $p_{\textrm{T}} ^2$ is taken to be the primary vertex (PV) of $\text {p}\text {p}$ interactions as described in Section 9.4.1 of Ref. [17]. The physics objects are the jets, clustered using the jet finding algorithm [18, 19] with the tracks assigned to candidate vertices as inputs, and the associated missing transverse momentum.

Jets are reconstructed from PF objects, clustered using the anti-$k_{\textrm{T}}$ algorithm [18, 19] with a distance parameter of $R=0.4$. The jet momentum is determined as the vectorial sum of all particle momenta in the jet, and is found from simulation to be, typically, within 5–10% of the true momentum over the entire $p_{\textrm{T}}$ spectrum and detector acceptance. Additional $\text {p}\text {p}$ interactions within the same or nearby bunch crossings can contribute additional tracks and calorimetric energy deposits, increasing the apparent jet momentum. To mitigate this effect, tracks identified as originating from pileup vertices are discarded and an offset correction based on the average amount of transverse energy in the event per unit area is applied to correct for the remaining contributions [20]. Jet energy corrections are derived from simulation studies so that the average measured energy of jets becomes identical to that of particle-level jets. However, the selective ECAL readout leads to a bias in the jet energy scale. In situ measurements of the momentum balance in dijet, photon + jet, Z + jet, and multijet events are used to determine any residual differences between the jet energy scale (JES) in data and in simulation, and appropriate corrections are made [21]. Additional selection criteria are applied to each jet to remove jets potentially dominated by instrumental effects or reconstruction failures. The jet energy resolution (JER) amounts typically to 15–20% at 30$\,\text {GeV}$, 10% at 100$\,\text {GeV}$, and 5% at 1$\,\text {TeV}$  [21].

The missing transverse momentum vector ${\vec p}_{\textrm{T}}^{\text {miss}}$ is computed as the negative vector sum of the transverse momenta of all the PF candidates in an event, and its magnitude is denoted as $p_{\textrm{T}} ^\text {miss}$  [22]. The ${\vec p}_{\textrm{T}}^{\text {miss}}$ is modified to include corrections to the energy scale of the reconstructed jets in the event. Anomalous high-$p_{\textrm{T}} ^\text {miss}$ events can be due to a variety of reconstruction failures, detector malfunctions, or noncollision backgrounds. Such events are rejected by event filters that are designed to identify more than 85–90% of the spurious high-$p_{\textrm{T}} ^\text {miss}$ events with a mistagging rate less than 0.1% [22].

A more detailed description of the CMS detector, together with a definition of the coordinate system used and the relevant kinematic variables, can be found in Ref. [23].

## Theoretical predictions

Theoretical predictions from Monte Carlo (MC) event generators at LO and NLO are used for comparison with measurements of the jet multiplicities as well as with the $p_{\textrm{T}}$ spectra in multijet final states.

We use the following predictions at LO:

- pythia 8 [24] (version 8.219) simulates LO $2\rightarrow 2$ hard processes. The parton shower is generated in a phase space ordered in transverse momentum and longitudinal momentum of the emitted partons, and the colored strings are hadronized using the Lund string fragmentation model. The CUETP8M1 [25] tune (with the parton distribution function (PDF) set NNPDF2.3LO [26]) gives the parameters for multiparton interactions (MPI).
- herwig++ [27] (version 2.7.1) simulates LO $2\rightarrow 2$ hard processes. The emitted partons in the parton shower follow angular ordering conditions, and the cluster fragmentation model is used to transform colored partons into observable hadrons. The CUETHppS1 [25] tune (with the PDF set CTEQ6L1 [28]) is applied.
- MadGraph 5_amc@nlo [4] (version 2.3.3) event generator, labeled MadGraph+Py8, is used in the LO mode, with up to four noncollinear high-$p_{\textrm{T}}$ partons included in the matrix element (ME), supplemented with parton showering and MPI using pythia 8 with the CUETP8M1 tune and merged according to the $k_{\textrm{T}}$-MLM matching procedure [29] with a matching scale of $10\,\text {GeV}$.
- MadGraph 5_amc@nlo [4] (version 2.6.3) event generator, labeled MadGraph+CA3, is used in the LO mode to generate up to four noncollinear high-$p_{\textrm{T}}$ partons included in the ME. The PB-method describes the DGLAP evolution as a process of successive branching processes. It has been shown [2, 3] that on an inclusive level, the DGLAP evolution is exactly reproduced, whereas the simulation of each branching determines the transverse momentum distribution obtained during evolution. The PB-TMDs are obtained from a fit to inclusive HERA deep-inelastic electron proton collision data [1]. Since the PB-TMDs come from a (forward) branching evolution, the initial-state parton shower (in a backward evolution) follows directly from the PB-TMD distributions, and therefore no free parameters are left for the initial-state shower. We use the TMD merging [30] procedure for combining the TMD parton shower with the ME calculations. The NLO PB-TMD set 2 [1] with $\alpha _\textrm{S} (m_{\text {Z}}) = 0.118$ is used. The inclusion of the transverse momentum $k_{\textrm{T}}$ and initial-state PB-TMD parton shower is performed with Cascade3  [5] (version 3.2.3). The initial-state parton shower follows the PB-TMD distribution, and has no free parameters left. The final-state radiation (since not constrained by TMDs) as well as hadronization is performed with pythia 6 (version 6.428) [31] with an angular ordering veto imposed. A merging scale value of 30$\,\text {GeV}$ is used, since it provides a smooth transition between ME and PS computations. MPI effects are not simulated.

At NLO, different theoretical predictions are used. The factorization and renormalization scales are set to half the sum over the scalar transverse momenta of all produced partons, $1/2 \sum H_{\textrm{T}}$. However, we have explicitly checked that the distributions do not change when choosing $1/4 \sum H_{\textrm{T}}$ or even $1/6 \sum H_{\textrm{T}}$. The uncertainty bands of the NLO predictions are determined from the variation of the factorization and renormalization scales by a factor of two up and down, avoiding the largest scale differences.

- MadGraph 5_amc@nlo [4] (version 2.6.3) interfaced with pythia 8 (version 8.306), labeled MG5_aMC+Py8 (jj), is used with MEs computed at NLO for the process $\text {p}\text {p}\rightarrow \textrm{jj}$. The NNPDF 3.0 NLO PDF set [32] is used and $\alpha _\textrm{S} (m_{\text {Z}})$ is set to 0.118. This calculation uses the CUETP8M1 tune to study the effect of MPI.
- MadGraph 5_amc@nlo [4] (version 2.6.3) interfaced with Cascade3 (version 3.2.2) [5], labeled MG5_aMC+CA3 (jj), is used with MEs computed at NLO for the process $\text {p}\text {p}\rightarrow \textrm{jj}$. The Herwig6 [33] subtraction terms are used in mc@nlo  [34, 35], since they are closest to the needs for applying PB-TMD parton densities, as described in Ref. [5]. The NLO PB-TMD set 2 [1] with $\alpha _\textrm{S} (m_{\text {Z}}) = 0.118$ is used. MPI are not simulated in this approach.
- MadGraph 5_amc@nlo [4] (version 2.6.3) interfaced with Cascade3, labeled MG5_aMC+CA3 (jjj) NLO, is used with MEs computed at NLO for the process $\text {p}\text {p}\rightarrow \textrm{jjj}$. The same PB-TMD distribution and parton shower calculation procedure as for MG5_aMC+CA3 (jj) are used.

In Table 1, the Monte Carlo event generators utilized in this analysis are summarized. Events generated by pythia 8, MadGraph+Py8, and herwig++ are passed through a full detector simulation based on Geant4  [36]. The simulated events are reconstructed the same way as the observed data events.

**Table 1:** Description of the simulated samples used in the analysis

Generator	PDF	ME	Tune
pythia 8 [24]	NNPDF 2.3 (LO) [26]	LO $2\rightarrow 2$	CUETP8M1 [25]
MadGraph+Py8 [4]	NNPDF 2.3 (LO) [26]	LO $2\rightarrow 2,3,4$	CUETP8M1 [25]
MadGraph+CA3 [4]	PB-TMD set 2 (NLO) [1]	LO $2\rightarrow 2,3,4$	–
herwig++ [27]	CTEQ6L1 (LO) [28]	LO $2\rightarrow 2$	CUETHppS1 [25]
MG5_aMC+Py8 (jj)	NNPDF 3.0 (NLO) [32]	NLO $2\rightarrow 2$	CUETP8M1 [25]
MG5_aMC+CA3 (jj)	PB-TMD set 2 (NLO) [1]	NLO $2\rightarrow 2$	–
MG5_aMC+CA3 (jjj)	PB-TMD set 2 (NLO) [1]	NLO $2\rightarrow 3$	–

**Table 2:** The integrated luminosity for each trigger sample considered in this analysis with the $p_{\textrm{T}}$ thresholds for HLT (PF) reconstruction

$p_{\textrm{T}} ^\text {HLT}$ ($\text {GeV}$)	140	200	260	320	400	450
$p_{\textrm{T}} ^\text {PF}$ ($\text {GeV}$)	196	272	362	430	548	592
$\mathcal {L} (\text {pb}^{-1})$	24.2	103	594	1770	5190	36300

## Data analysis

The measured data samples recorded in 2016, corresponding to an integrated luminosity of $36.3{\,\text {fb}^{-1}}$, were collected with single-jet HLTs. For each single-jet HLT at least one jet with $p_{\textrm{T}}$ higher than the $p_{\textrm{T}}^{\textrm{HLT}}$ trigger threshold is required. All triggers, except the one with the highest $p_{\textrm{T}}^{\textrm{HLT}}$ threshold, were prescaled. We consider events only if the leading jet, reconstructed with the PF algorithm, can be matched with an HLT jet. In Table 2, the integrated luminosity $\mathcal {L}$ for each trigger is shown. The trigger efficiency ($>99.5\%$) is estimated using triggers with lower $p_{\textrm{T}}^{\textrm{HLT}}$ thresholds; for the trigger with lowest $p_{\textrm{T}}^{\textrm{HLT}}$ threshold a tag-and-probe [14] method is used to determine the $p_{\textrm{T}}$ threshold. To address the issue of trigger inefficiency resulting from prefiring (discussed in Sect. 2), the simulated event samples are corrected on an event-by-event basis prior to unfolding. Maps of the prefiring probability in the region of $2.0< |\eta | < 3.0$ are utilized, taking into account the $p_{\textrm{T}}$ and $\eta$ of the jets. The total event weight is then calculated as the product of the nonprefiring probability of all jets. For each $p_{\textrm{T}}$ and $\eta$ bin in the prefiring maps, the uncertainty per jet is determined by taking the maximum value between 20% of the prefiring probability and the statistical uncertainty.

The jets are corrected using the JES correction procedure in CMS [21], and an additional smoothing procedure (described in Ref. [37]) is applied to the JES correction. The simulated samples are corrected to take into account the JER by spreading the $p_{\textrm{T}}$ of the jets according to the resolution extracted from data. Jets reconstructed in regions of the detector corresponding to noisy towers in the calorimeters are excluded from the measurement [38].

The pileup profile used in simulation is corrected to reproduce the one in data.

### Event selection

Each event is required to have a reconstructed PV. The PV must satisfy $|z_\textrm{PV} |<24\,\text {cm}$ and $\rho _\textrm{PV}<2\,\text {cm}$, where $z_\textrm{PV}$ ($\rho _\textrm{PV}$) is the longitudinal (radial) distance from the nominal interaction point.

All events that contain jets clustered using the anti-$k_{\textrm{T}}$ algorithm [18, 19] with a distance parameter of $R=0.4$ and reconstructed with $|\eta |<3.2$ and transverse momentum $p_{\textrm{T}} >20\,\text {GeV}$ are preselected. From these events, the ones with at least a pair of jets with $p_{\textrm{T1}} >200\,\text {GeV}$, $p_{\textrm{T2}} >100\,\text {GeV}$ and $|y^{{1,2}} |<2.5$ are selected (events with one of the leading two jets with $|y |>2.5$ are vetoed). Additional jets must have $p_{\textrm{T}} > 50\,\text {GeV}$ and $|y | < 2.5$. In addition, jets must satisfy quality criteria based on the jet constituents, in order to reject misidentified jets [39]. The selected events must have a missing transverse energy fraction smaller than 0.1 (more details in Sect. 4.3). The selection at particle-level includes only the $p_{\textrm{T}}$ and $|y |$ constraints on the jets.

### Observables and phase space

In the following, the observables will be described at particle-level. The particle-level is defined after all the partons have hadronized to form stable particles with a proper lifetime above 10$\,\text {mm}$/c.

The exclusive jet multiplicity ($N_{\text {jets}}$) of up to 6 jets (inclusive for $N_{\text {jets}} \ge 7$), in three bins of $p_{\textrm{T1}}$ ($200< p_{\textrm{T1}} < 400$, $400< p_{\textrm{T1}} < 800$, and $p_{\textrm{T1}} > 800\,\text {GeV}$) and for three different regions in $\varDelta \phi _{1,2}$ ($0< \varDelta \phi _{1,2} < 150$, $150< \varDelta \phi _{1,2} < 170$, $170< \varDelta \phi _{1,2} < 180^\circ$) is measured:

1 $$\begin{aligned} \frac{\text {d}\sigma _\text {dijet}}{\text {d}N^\textrm{i}_\text {jets}\text {d}p^\textrm{j}_\textrm{T1}\text {d}(\varDelta \phi _{1,2} ^\textrm{k})} \end{aligned}$$

where $\textrm{i},\textrm{j},\textrm{k}$ corresponds to the binning in $N_{\text {jets}}$, $p_{\textrm{T1}}$, and $\varDelta \phi _{1,2}$. We measure exclusive jet multiplicities since we are interested in how the hard and soft multijet radiation is modeled by including higher-order corrections and parton shower effects.

In addition, the differential cross section as a function of the $p_{\textrm{T}}$ of each of the four leading jets are measured:

2 $$\begin{aligned} \frac{\text {d} \sigma _{\text {p}\text {p}\rightarrow \textrm{jj}}}{\text {d} p_{\textrm{T1}}}, \quad \frac{\text {d} \sigma _{\text {p}\text {p}\rightarrow \textrm{jj}}}{\text {d} p_{\textrm{T2}}}, \quad \frac{\text {d} \sigma _{\text {p}\text {p}\rightarrow \textrm{jjj}}}{\text {d} p_{\textrm{T3}}}, \quad \frac{\text {d} \sigma _{\text {p}\text {p}\rightarrow \textrm{jjjj}}}{\text {d} p_{\textrm{T4}}}, \end{aligned}$$

where $\sigma _{\text {p}\text {p}\rightarrow \textrm{jj}}$, $\sigma _{\text {p}\text {p}\rightarrow \textrm{jjj}}$, $\sigma _{\text {p}\text {p}\rightarrow \textrm{jjjj}}$ correspond to the dijet, three-jet, and four-jet cross sections in proton–proton collision.

**Fig. 1:**
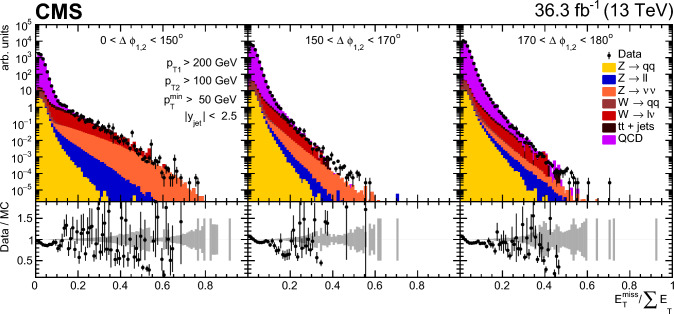
Distribution of $E_{\textrm{T}}^{\text {miss}}/\sum {E_{\textrm{T}}}$ for data and simulated jet production for three regions of $\varDelta \phi _{1,2}$. Shown are the contributions from QCD, $\text {W}/\text {Z}$ and ${\text {t}{}\overline{\text {t}}}$ events. The main contributions of events with large $E_{\textrm{T}}^{\text {miss}}$ in the final state come from processes like $\text {Z}\rightarrow \upnu \overline{\upnu }$ and $\text {W}\rightarrow \text {l}\upnu$. The data (MC prediction) statistical uncertainty is shown as a vertical line (grey shaded bar in the ratio)

### Background treatment

To remove background contributions from ${\text {t}{}\overline{\text {t}}} +\text {jets}$, $\text {W}/\text {Z}+\text {jets}$, $\text {Z}\rightarrow \upnu \overline{\upnu }$ and $\text {W}\rightarrow \text {l}\upnu$, events with $E_{\textrm{T}}^{\text {miss}}/\sum {E_{\textrm{T}}} > 0.1$ are rejected. The missing transverse energy is calculated from $p_{\textrm{T}} ^\text {miss}$ and the sum $\sum {E_{\textrm{T}}}$ runs over all PF objects in the event. Less than 1% of the events are rejected in the whole sample. For $N_{\text {jets}} = 2$ this constraint becomes important and the backgournd contribution is reduced from 20% to the percent level for $p_{\textrm{T1}} > 800\,\text {GeV}$ in the low $\varDelta \phi _{1,2}$. In Fig. 1, the measured fraction $E_{\textrm{T}}^{\text {miss}}/\sum {E_{\textrm{T}}}$ is compared with the simulation of signal and background processes in bins of $\varDelta \phi _{1,2}$.

**Fig. 2:**
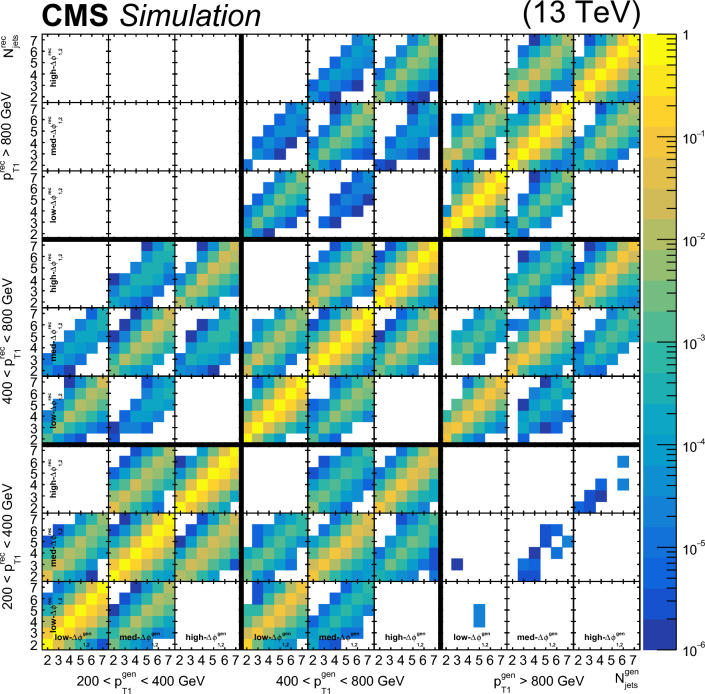
Probability matrix (condition number: 3.0) for the jet multiplicity distribution constructed with the MadGraph+Py8 sample. The global 3$\times$3 sectors (separated by the thick black lines) correspond to the $p_{\textrm{T1}}$ bins, indicated by the labels in the x (lower) and y (left) axes; the smaller 3$\times$3 structures correspond to the $\varDelta \phi _{1,2}$ bins, indicated in the leftmost row and lowest column, the x(y) axis of these $\varDelta \phi _{1,2}$ cells corresponds to the jet multiplicity at particle (detector) level. The z axis covers a range from $10^{-6}$ to 1 indicating the probability of migrations from the particle-level bin to the corresponding detector-level bin. The lower right corner of the matrix, which describes (extreme) migrations between hight $p_{\textrm{T1}}$ at particle level and low $p_{\textrm{T1}}$ at dectector level, is characterized by statistical fluctuations

### Correction for detector effects

The measured cross sections are corrected for misidentified events, detector resolution, and inefficiencies for comparison with particle-level predictions through the procedure of unfolding. A response matrix (RM) mapping the “true” distribution onto the measured one is constructed using simulated MC samples from pythia 8, MadGraph+Py8, and herwig++. The MadGraph+Py8 sample, which has the smallest statistical uncertainty, is used as default for constructing the RM, whereas the herwig++ and pythia 8 samples are used to investigate the model dependence. The RM is constructed by matching the detector and particle-level distributions. If events (or jets) cannot be matched, they contribute to the background or inefficiency distributions. For the jet multiplicity, the dijet system (leading and subleading jets) is matched if the jets coincide within $\varDelta R < 0.2$ (half of the jet radius of $R = 0.4$). For the $p_{\textrm{T}}$ distributions, jets are matched with $\varDelta R < 0.2$, and from the matched candidates the one highest in $p_{\textrm{T}}$ is selected (only events with at least two jets are considered in the matching). The TUnfold (version 17.9) package [40] is used to perform the unfolding.

The jet multiplicity is obtained by matrix inversion:

3 $$\begin{aligned} \textbf{A}\alpha + \beta = \gamma \end{aligned}$$

where $\gamma (\alpha )$ is the distribution at detector (particle) level, $\textbf{A}$ is the probability matrix (PM), and $\beta$ is the contribution from background (or noise). The PM is obtained by normalizing the RM to the particle-level axis (row-by-row normalization), and describes the probability that a particle-level jet (or event) generated in a bin is reconstructed in (migrates to) another bin at detector-level. The condition number of the PM is defined as the ratio between the largest and smallest singular value of the matrix. The condition number of the PM for the jet multiplicity is 3.0, and matrix inversion is possible.

For the $p_{\textrm{T}}$ distributions, the condition number of the PM is 4.9, and least-square minimisation is applied:

4 $$\begin{aligned} \chi ^2&= \min _{\alpha } \left[ (\gamma - \beta - \textbf{A} \alpha )^\intercal \, {\textbf{V}_{\gamma \gamma }}^{-1}\, (\gamma - \beta - \textbf{A}\alpha ) \right] , \end{aligned}$$

where $\textbf{V}_{\gamma \gamma }$ represents the statistical covariance matrix of $\gamma$ and $\beta$, with twice the number of bins at detector level compared to particle-level. No additional regularization is needed.

**Fig. 3:**
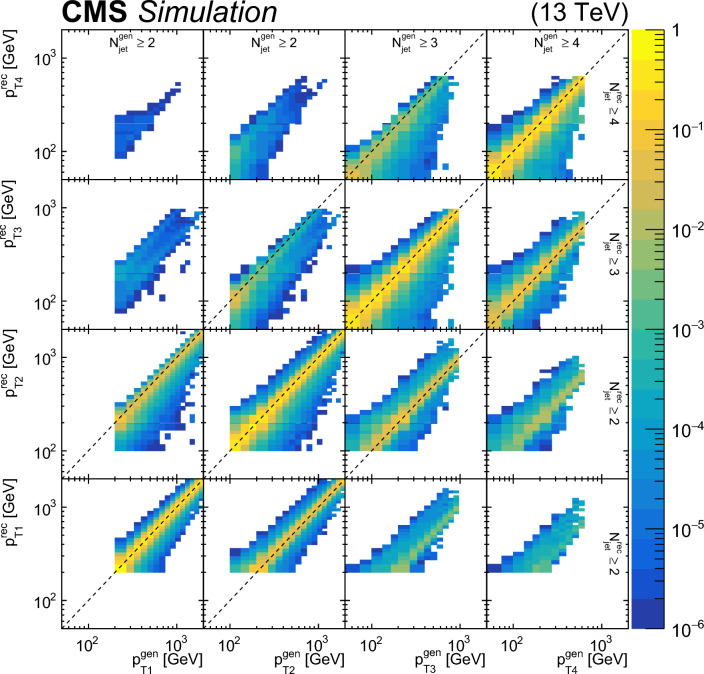
Probability matrix (condition number: 4.9) for the $p_{\textrm{T}}$ of the four leading jets constructed with the MadGraph+Py8 sample. The global 4$\times$4 sectors correspond to the $p_{\textrm{T}}$ distributions each of the first four jets, the x axis corresponds to the particle (gen) level and y axis corresponds to the detector (rec) level. The z axis covers a range from $10^{-6}$ to 1 indicating the probability of migrations from the particle-level bin to the corresponding detector-level bin

In Figs. 2 and 3, the PMs are shown for the multiplicity distributions and the $p_{\textrm{T}}$ distributions of the first four jets.

### Uncertainties

**Fig. 4:**
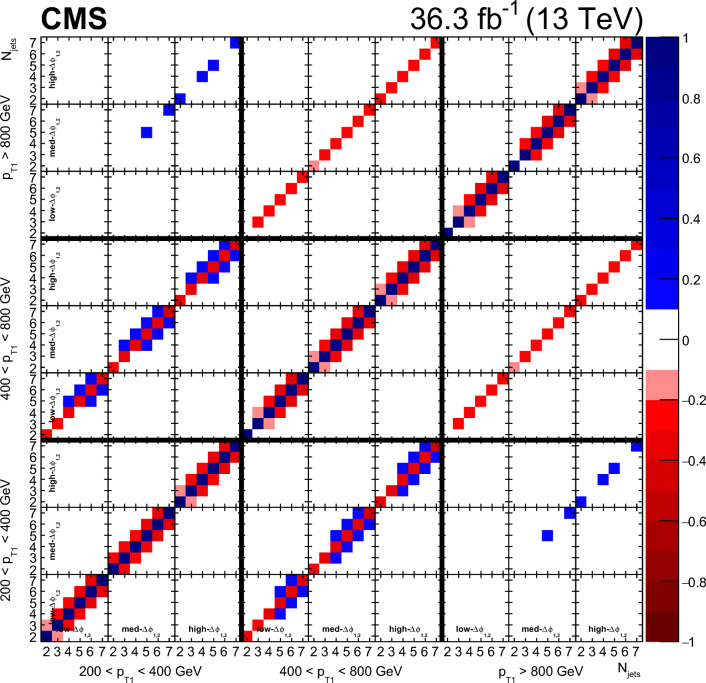
Correlation matrix at the particle-level for the jet multiplicity distribution. It contains contributions from the data and from the limited-size MadGraph+Py8 sample. The global 3$\times$3 sectors (separated by the thick black lines) correspond to the $p_{\textrm{T1}}$ bins, indicated by the labels next to the x (lower) and y (left) axes; the smaller 3$\times$3 structures correspond to the $\varDelta \phi _{1,2}$ bins, indicated in the leftmost row and lowest column, the x and y axes correspond to the jet multiplicity. The z axis covers a range from $-\,1$ to 1 indicating the correlations in blue shades and anticorrelations in red shades, the values between $-\,0.1$ and 0.1 are represented in white

**Fig. 5:**
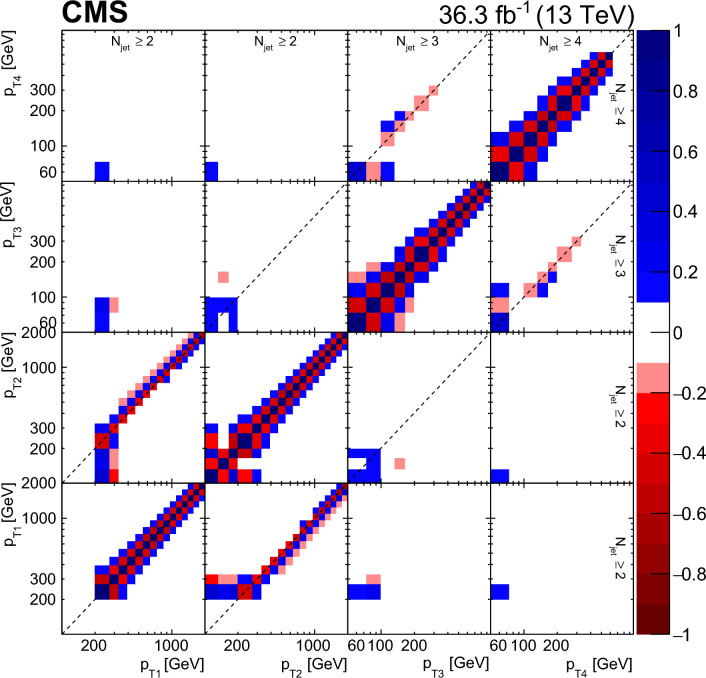
Correlation matrix for the particle-level $p_{\textrm{T}}$ of the four leading jets. It contains contributions from the data and from the limited-size MadGraph+Py8 sample. Here each one of the 4$\times$4 sectors corresponds to one of the $p_{\textrm{T}}$ spectra measured, indicated by the x and y axis labels. The z axis covers a range from $-\,1$ to 1 indicating the correlations in blue shades and anticorrelations in red shades, the values between $-\,0.1$ and 0.1 are represented in white

The statistical uncertainties in the measured spectra and response matrices are propagated to the final results. In Figs. 4 and 5, the statistical correlations are shown for the jet multiplicity and for the $p_{\textrm{T}}$ spectra of the four leading jets.

The systematic uncertainties originate from the following sources:

- JES: The JES uncertainty is estimated from variations of one standard deviation in the JES corrections applied to data (at detector-level) and repeating the whole unfolding procedure for each variation.
- JER: The JER uncertainty is estimated by varying the resolution by one standard deviation in the simulated sample, and repeating the unfolding for each variation.
- Integrated luminosity: The uncertainty in the integrated luminosity is 1.2% [41] and is applied as a global scaling factor to the cross section.
- Pileup: The pileup distribution in the simulated samples is reweighted to match that of the data. The corresponding uncertainty is estimated by varying the total inelastic cross section by $\pm \,5\%$ [42], affecting the measurement by less than 1%.
- Trigger prefiring uncertainty: The trigger prefiring uncertainty is estimated by varying the correction applied to the simulated samples and repeating the unfolding for each variation, resulting in an uncertainty of 1–3%.
- Model uncertainty: The model uncertainty is estimated by varying the distributions of the factorization and renormalization scales in the MC sample such as to still describe the detector level distributions. Additionally background and inefficiencies are varied separately by 15%. The final uncertainty is the quadratic sum of each of the uncertainties. It was validated with a set of closure tests performed using pythia 8 and herwig++ samples as pseudodata unfolded with MadGraph+Py8 RM. This uncertainty ranges from 1 to 7%.

The total systematic uncertainty is obtained by adding all the systematic uncertainties in quadrature, assuming independent sources.

**Fig. 6:**
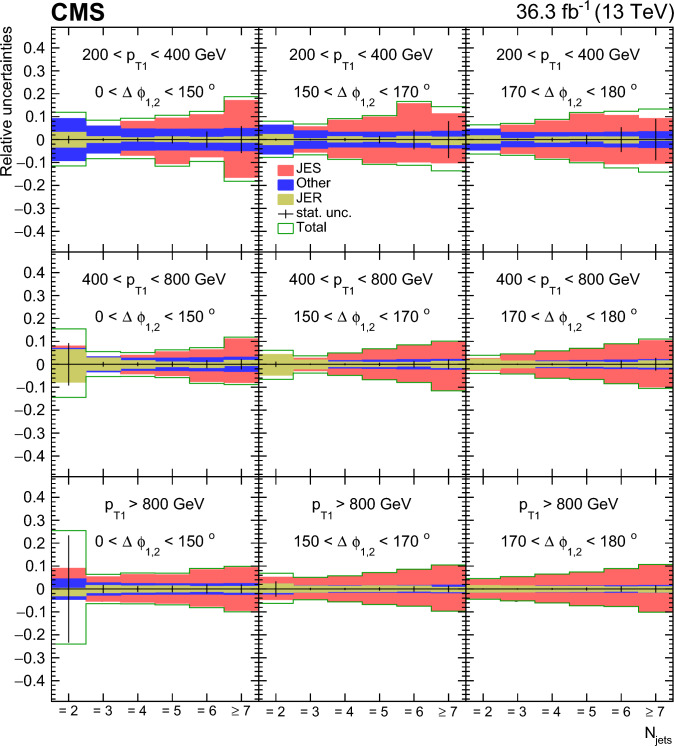
Relative uncertainties for JES, JER, “Other” and total statistical uncertainty for the jet multiplicity distribution in bins of $p_{\textrm{T1}}$ and $\varDelta \phi _{1,2}$. Here “Other” indicates luminosity, pileup, prefiring, and model uncertainty

In Fig. 6, the relative uncertainties for the jet multiplicity in bins of $p_{\textrm{T1}}$ and $\varDelta \phi _{1,2}$ are shown. The dominant uncertainty is JES. The total statistical uncertainty (stat. unc.) is mainly driven by the limited event counts in data. The total experimental uncertainty (Total) is typically about 10–15%.

**Fig. 7:**
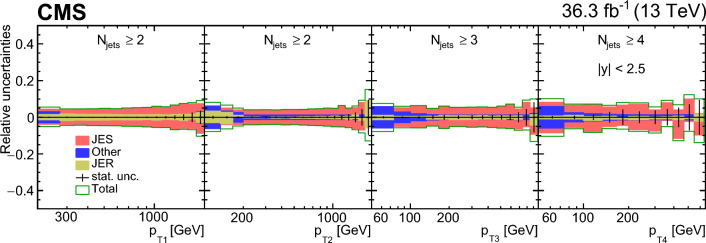
Relative uncertainties for JES, JER, “Other” and total statistical uncertainty for the $p_{\textrm{T}}$ distributions of the four leading jets. Here “Other” indicates luminosity, pileup, prefiring, and model uncertainty

In Fig. 7, the relative uncertainties as a function of the jet $p_{\textrm{T}}$ for the four leading jets are shown. The dominant uncertainty is JES. The measurement is limited by the systematic uncertainty and the total experimental uncertainty ranges from 5 to 10%.

## Results

The phase space of the measurements at particle-level (particles with a proper lifetime above 10$\,\text {mm}$/c) is defined by jets clustered using the anti-$k_{\textrm{T}}$ algorithm [18, 19] with a distance parameter of $R=0.4$ within $|y | < 3.2$. Events are selected if the two highest $p_{\textrm{T}}$ jets with $p_{\textrm{T1}} > 200\,\text {GeV}$, $p_{\textrm{T2}} > 100\,\text {GeV}$ have $|y |< 2.5$ (i.e., events are not counted where one of the leading jets has $|y |> 2.5$). For the additional jets $p_{\textrm{T}} >50\,\text {GeV}$ and $|y |< 2.5$ is required. All predictions (LO and NLO) are normalized to the measured dijet cross section in the measurement phase space, with the normalization factors explicitly given in the figures.

### Jet multiplicity distribution

**Fig. 8:**
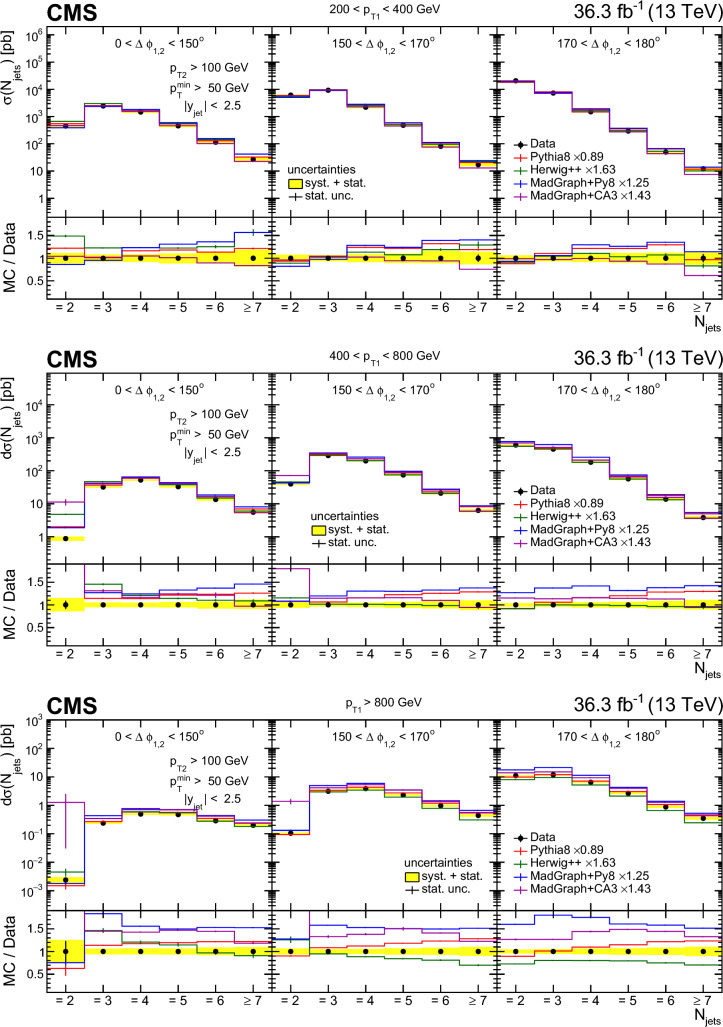
Differential cross section as a function of the exclusive jet multiplicity (inclusive for 7 jets) in bins of $p_{\textrm{T1}}$ and $\varDelta \phi _{1,2}$. The data are compared with LO predictions of pythia 8, herwig++, MadGraph+Py8 and MadGraph+CA3. The predictions are normalized to the measured dijet cross section using the scaling factors shown in the legend. The vertical error bars correspond to the statistical uncertainty, the yellow band shows the total experimental uncertainty

The multiplicity of jets with $$p_{\textrm{T}} > 50\,\text {GeV} $$ in $$|y |< 2.5 $$ is measured for various regions of the transverse momentum of the leading jet, $$p_{\textrm{T1}}$$, and the azimuthal angle $$\varDelta \phi _{1,2}$$ between the two leading jets as shown in Fig. 8.

As a characterization of the jet multiplicity we compare the production rate for 3 jets with the one for 7 jets. In the region of low $p_{\textrm{T1}}$ ($200< p_{\textrm{T1}} < 400\,\text {GeV}$), a large number of additional jets is observed at low $\varDelta \phi _{1,2}$ ($0< \varDelta \phi _{1,2} < 150^\circ$), the production rate between 3 and 7 jets changes by two orders of magnitude. In the large-$\varDelta \phi _{1,2}$ region ($170< \varDelta \phi _{1,2} < 180^\circ$), where the leading jets are nearly back-to-back, the production rate for 3 to 7 jets changes by three orders of magnitude. We note that even in this back-to-back region a large number of additional jets are observed that do not contribute significantly to the momentum imbalance of the two leading jets.

In the region of large $p_{\textrm{T1}}$ ($p_{\textrm{T1}} >800\,\text {GeV}$), we observe that the rate of additional jets at low $\varDelta \phi _{1,2}$ is essentially constant and the rate between 3 and 7 jets only shows small changes, indicating that many jets participate in the compensation of the $\varDelta \phi _{1,2}$ decorrelation. In the large-$\varDelta \phi _{1,2}$ region ($170< \varDelta \phi _{1,2} < 180^\circ$), the rate between 3 and 7 jets changes by less than 2 orders of magnitude, in contrast to the low-$p_{\textrm{T1}}$ region. A multiplicity of more than two or three additional jets at large $p_{\textrm{T1}}$ is observed in all regions of $\varDelta \phi _{1,2}$.

In Fig. 8, predictions from the LO $2\rightarrow 2$ generators pythia 8 and herwig++ including parton showering and MPI are shown. The shape of the prediction coming from pythia 8 is different from what is observed in the measurement. The shape of the prediction from herwig++ agrees rather well with the measurement, especially in the large $\varDelta \phi _{1,2}$ region. The difference between pythia 8 and herwig++ in jet multiplicity comes from the different treatment of the parton shower. In addition the predictions from MadGraph+Py8 and MadGraph+CA3 with additional noncollinear high-$p_{\textrm{T}}$ partons, supplemented with different parton showering approaches and MPI are shown. We find that the merged prediction from MadGraph+Py8 does not agree in shape with the measurement, whereas the MadGraph+CA3 merged prediction (for $N_{\text {jets}} > 2$ and $p_{\textrm{T1}} < 800 \,\text {GeV}$) does, similarly to the case of herwig++.

**Fig. 9:**
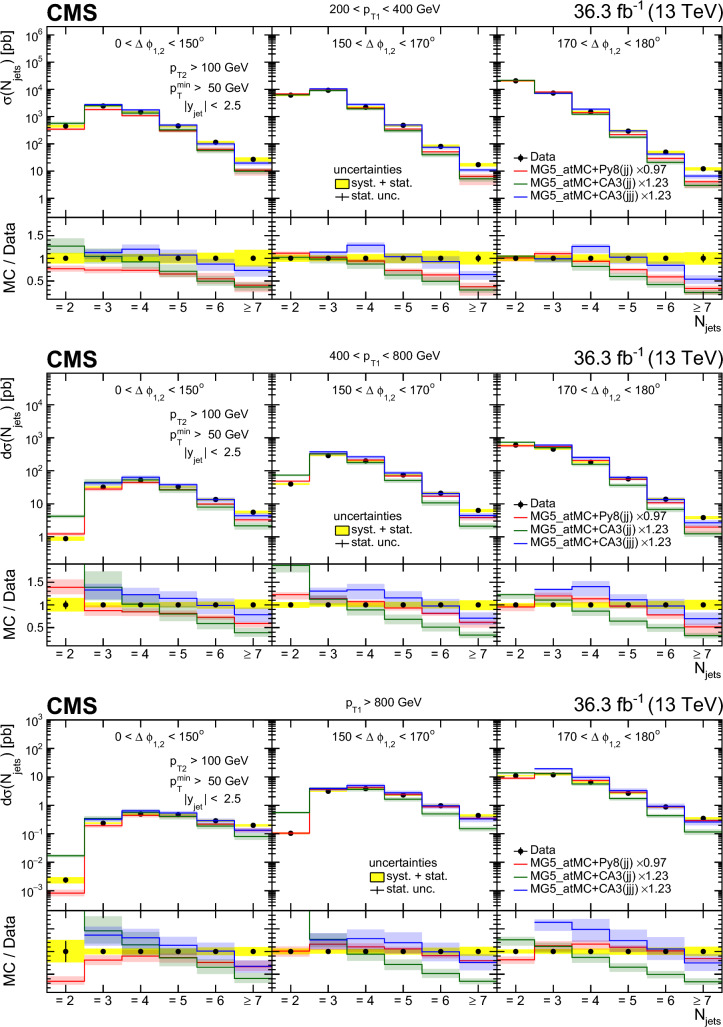
Differential cross section as a function of the exclusive jet multiplicity (inclusive for 7 jets) in bins of $p_{\textrm{T1}}$ and $\varDelta \phi _{1,2}$. The data are compared with NLO dijet predictions MG5_aMC+Py8 (jj) and MG5_aMC+CA3 (jj) as well as the NLO three-jet prediction of MG5_aMC+CA3 (jjj). The vertical error bars correspond to the statistical uncertainty, the yellow band shows the total experimental uncertainty. The shaded bands show the uncertainty from a variation of the renormalization and factorization scales. The predictions are normalized to the measured inclusive dijet cross section using the scaling factors shown in the legend

The calculations with NLO MEs matched with parton shower compared with the measurements are shown in Fig. 9. The uncertainty bands of the predictions come from the variation of the factorization and renormalization scales by a factor of two up and down, avoiding the largest scale differences. The normalization of the MG5_aMC+Py8 (jj) calculation is in reasonable agreement with the measured cross section even for three jets. For higher jet multiplicities the prediction falls below the measurement. MG5_aMC+CA3 (jj) predicts a smaller cross section for more than three jets compared with the measurement. The MG5_aMC+CA3 (jjj) NLO calculation (using the same normalization factor as for MG5_aMC+CA3 (jj)) predicts a larger three- and four-jet cross section, whereas the higher jet multiplicities are still underestimated.

### Transverse momenta of the four leading jets

**Fig. 10:**
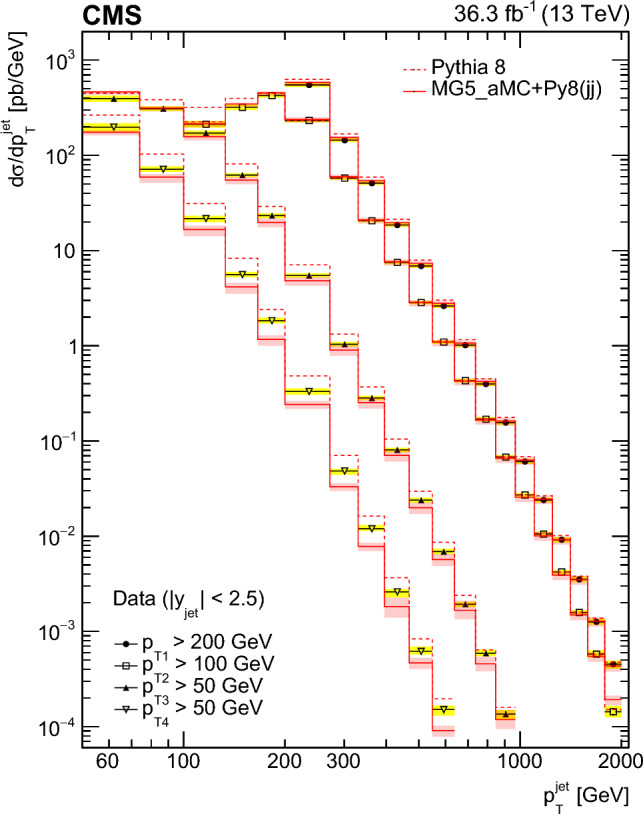
Transverse momenta of the four leading jets, with the yellow band representing the total experimental uncertainty. The data are compared with LO (pythia 8) and NLO (MG5_aMC+Py8) predictions. The red band in the NLO prediction represents the renormalization and factorization scale uncertainty

The measured differential jet cross section as a function of the jet transverse momentum, $p_{\textrm{T}}$, for the four leading $p_{\textrm{T}}$ jets is shown in Fig. 10 and compared with the predictions of pythia 8 and MG5_aMC+Py8 (jj). The $p_{\textrm{T}}$ values of the two leading-$p_{\textrm{T}}$ jets reaches up to 2$\,\text {TeV}$ and the third and fourth jets $p_{\textrm{T}}$ reaches about 1$\,\text {TeV}$. We observe that the shape of the $p_{\textrm{T}}$ spectrum for the third and fourth leading jets is qualitatively similar to the one of the two leading jets, whereas the cross section is different. The rise of the cross section for the second jet between 100$\,\text {GeV}$ and 200$\,\text {GeV}$ is a consequence of the higher $p_{\textrm{T}}$ requirement ($p_{\textrm{T1}} > 200\,\text {GeV}$) applied to the leading jet in the event selection.

**Fig. 11:**
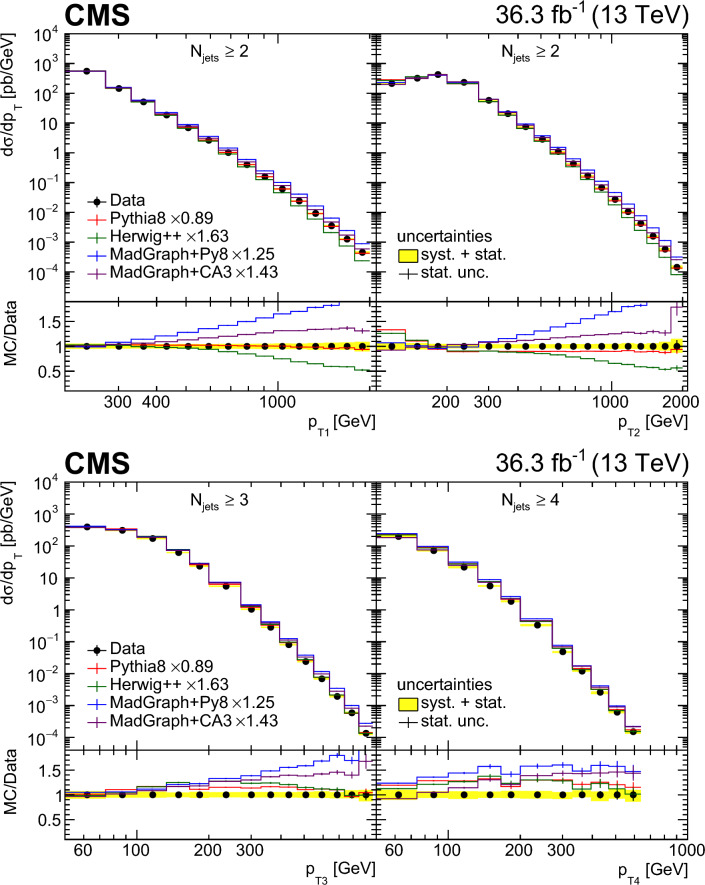
Transverse momentum distributions of the four leading jets. The transverse momentum of the leading and subleading (third and fourth leading) $p_{\textrm{T}}$ jets from left to right is shown in the upper (lower) figure. The data are compared with LO predictions of pythia 8, herwig++, MadGraph+Py8 and MadGraph+CA3. The predictions are normalized to the measured dijet cross section using the scaling factors shown in the legend. The vertical error bars correspond to the statistical uncertainty, the yellow band shows the total experimental uncertainty

In Fig. 11, the measured differential cross section as a function of the $p_{\textrm{T}}$ for the four leading jets is shown and compared with LO predictions (using the same normalization factors as in Fig. 8). Only the prediction of pythia 8 is able to describe reasonably well the measurement in shape, except for the region $p_{\textrm{T2}} < 200\,\text {GeV}$. Among the LO calculations, pythia 8 and herwig++ predict a rather flat distribution for the third and fourth jet, whereas the other predictions are different in shape. The predictions from herwig++ are not in agreement in shape and rate with the measurements, the differences are up to 50% for the leading and subleading jets at large $p_{\textrm{T}}$. The prediction from MadGraph+Py8 gives a significantly different shape for the $p_{\textrm{T}}$ spectrum for the first 3 jets. Comparing the merged predicitons, MadGraph+CA3 gives a significant improvement in the description of the shape of the $p_{\textrm{T}}$ distribution for the three leading jets, whereas the description of the distribution of the fourth jet is similar to the one with MadGraph+Py8.

**Fig. 12:**
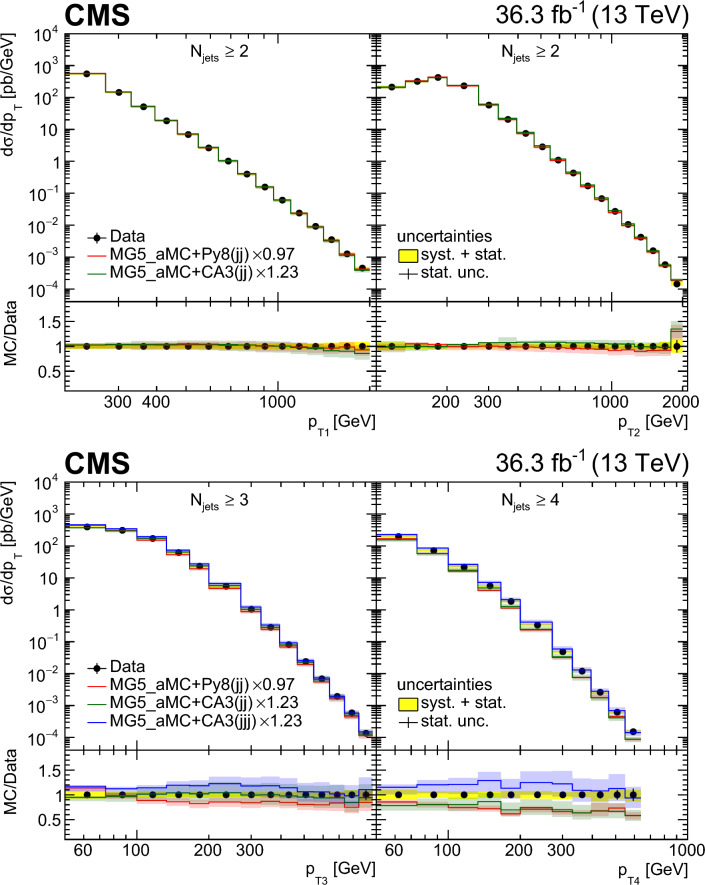
Transverse momentum distributions of the four leading jets. The transverse momentum of the leading and subleading (third and fourth leading) $p_{\textrm{T}}$ jets from left to right is shown in the upper (lower) figure. The data are compared with NLO predictions MG5_aMC+Py8 (jj) and MG5_aMC+CA3 (jj) as well as the NLO three-jet prediction of MG5_aMC+CA3 (jjj). The vertical error bars correspond to the statistical uncertainty, and the yellow band to total uncertainty of the measurement. The bands show the uncertainty from a variation of the renormalization and factorization scales. The predictions are normalized to the measured dijet cross section using the scaling factors shown in the legend

The predictions obtained with NLO MEs are shown in Fig. 12 using the same normalization factors as in Fig. 9. The uncertainty bands of the predictions come from the variation of the factorization and renormalization scales by a factor of two up and down, avoiding the largest scale differences. The predictions of MG5_aMC+Py8 (jj) and MG5_aMC+CA3 (jj) describe the normalization and the shape of the first three jets rather well, whereas for the fourth jet (which comes from the parton shower) falls below the measurement by 20–30%. The prediction of MG5_aMC+CA3 (jjj) describes the third and fourth jets rather well within uncertainties (predictions for the first and second jet are meaningless for MG5_aMC+CA3 (jjj) and therefore not shown). The calculations using PB-TMDs together with NLO MEs in the MC@NLO frame provide a good description of jet measurements over a wide range in transverse momentum and jet multiplicities without tunable parameters in the initial-state parton shower.

## Summary

A study of multijet events has been performed in proton–proton collisions at a center-of-mass energy of 13$\,\text {TeV}$ using data collected with the CMS detector corresponding to an integrated luminosity of $36.3{\,\text {fb}^{-1}}$. The measurements are performed by selecting a dijet system containing a jet with $p_{\textrm{T}} > 200\,\text {GeV}$ and a subleading jet with $p_{\textrm{T}} >100\,\text {GeV}$ within $|y | < 2.5$.

For the first time, the jet multiplicity in bins of the leading jet $p_{\textrm{T}}$ and the azimuthal angle difference between the two leading jets, $\varDelta \phi _{1,2}$, is measured. The jet multiplicity distributions show that even in the back-to-back region of the dijet system, up to seven jets are measurable. The differential production cross sections are measured for the highest $p_{\textrm{T}}$ jets up to the $\,\text {TeV}$ scale.

The measurement of the differential cross section as a function of the jet $p_{\textrm{T}}$ for the four highest $p_{\textrm{T}}$ jets is an important reference for QCD multijet cross section calculations, and especially for the simulations including parton showers for higher jet multiplicity.

The measured multiplicity distribution of jets with $p_{\textrm{T}} > 50\,\text {GeV}$ and $|y | < 2.5$ is not well described by the leading order MadGraph +pythia 8 simulation. However, in the back-to-back region herwig++ and MadGraph + Cascade3 provide a better description of the shape of the jet multiplicity. The measured differential cross section as a function of the transverse momentum of the four leading $p_{\textrm{T}}$ jets is not described by any of the LO predictions either in normalization or in shape. However, MadGraph + Cascade3 describes the shape of the distribution better than MadGraph +pythia 8.

The predictions using dijet NLO matrix elements, MG5_aMC+pythia 8 (jj) and MG5_aMC+ Cascade3 (jj) describe the lower multiplicity regions, as well as the transverse momenta of the leading jets, reasonably well. The three-jet NLO calculation MG5_aMC+ Cascade3 (jjj) describes very well the cross section of the third and fourth jets.

The measurements presented here provide stringent tests of theoretical predictions in the perturbative high-$p_{\textrm{T}}$ and high-jet multiplicity regions. Although the higher jet multiplicities are not described with either parton shower approach, it is interesting that the lower jet multiplicity cross section is described satisfactorily with NLO dijet calculations supplemented with PB-TMDs and TMD parton shower with fewer tunable parameters than in the case with conventional parton showers.

The measured observables and its statistical correlations are provided in HEPData [43] as tabulated results.

## Data Availability

This manuscript has no associated data or the data will not be deposited. [Authors’ comment: Release and preservation of data used by the CMS Collaboration as the basis for publications is guided by the CMS policy as stated in https://cms-docdb.cern.ch/cgibin/PublicDocDB/RetrieveFile?docid=6032 &filename=CMSDataPolicyV1.2.pdf &version=2 CMS data preservation, re-use and open access policy.]
